# Fit-for-purpose Psychological Interventions to Support the Well-Being of Autistic Adults: A Systematic Review

**DOI:** 10.1177/23969415261436238

**Published:** 2026-05-15

**Authors:** Michele Deakin, Lorna G. Hamilton, Brett Heasman, Stephanie Petty

**Affiliations:** School of Education, Language and Psychology, 41872 York St John University, York, UK

**Keywords:** Autism, wellbeing, systematic review, psychological interventions, outcome measures

## Abstract

**Background and aims:**

There are high levels of co-occurring mental health diagnoses reported for autistic adults, yet research into psychological interventions that support wellbeing is only just emerging. This mixed-methods systematic review characterized all available psychological interventions for autistic adults, the measures used to evaluate their effectiveness, the extent to which the interventions have been designed or adapted for autistic people, and the elements theorized to contribute to wellbeing.

**Methods:**

A protocol was registered with PROSPERO (CRD42023385232) before commencement. The review used Preferred Reporting Items for Systematic Reviews and Meta-Analyses guidelines. A Patient and Public Involvement process sought input from an expert panel of autistic counselors who work with autistic people to develop the search strategy. All study designs were included in the review to meet the objective of summarizing the available evidence since 2012, the point at which National Institute for Health and Care Excellence introduced Clinical Guidelines for working with autistic adults in the United Kingdom. Screening was performed on 100% of the records by two independent reviewers. Risks of bias that can occur in autism research specifically were assessed. Quality appraisal was undertaken using the *Quality Assessment Tool for Studies with Diverse Designs*. Mixed-methods and qualitative syntheses were used to summarize the findings.

**Main contribution:**

A total of 4,186 papers were identified from the literature search, resulting in 69 papers meeting the criteria for inclusion. The majority (*n* = 42) were pilot or exploratory designs. Few studies identified how adaptations to existing therapeutic interventions affected wellbeing outcomes. Similarly, few studies described the theorized mechanisms of change in reported wellbeing or designed interventions specifically for autistic people.

**Conclusions:**

There are few empirical studies of psychological interventions for autistic adults, and imprecise definitions of what wellbeing means for autistic people demonstrate conceptual and methodological ambiguity. Further discussion to agree community-based prioritization of the relevance of mental health outcomes is required. A minority of studies (*n* = 29) used outcome measures designed for, or standardized with, autistic people. The most common therapeutic interventions were based on mindfulness or cognitive-behavioral therapies, suggesting limited choice of evidence-based psychological interventions for autistic people and practitioners.

**Implications:**

Research into improving the wellbeing of autistic adults is still at an early stage of development, principally focused on assessing the feasibility of interventions. Current intervention studies are reliant on outcome measures with unknown suitability to evaluate meaningful change for autistic adults. This review provides an essential point of reference for clinical researchers and practising clinicians. A list of outcome measures that have been validated for use with autistic people is shared as a finding. The findings add to literature advocating for neurodiversity-affirmative clinical and research practices, which incorporate autistic phenomenology and recommend a critical approach towards psychological interventions.

High levels of co-occurring mental health diagnoses are reported for autistic adults, with 79% of people suggested to meet the diagnostic criteria for at least one mental health condition over their lifetime (Lever & Geurts, 2016). Improving mental health support is the highest research priority for autistic people (Autistica, 2019; Cage et al., 2024). Yet difficulties remain with accurately describing autistic people's mental health experiences for three main reasons. First, autistic wellbeing is often conceptualized through the lens of neuronormative models (Lam et al., 2021; Petty et al., 2023b), such that being “well” often means being “typical” (Milton & Sims, 2016). For example, experiences of loneliness can be understood in limited ways because frameworks tend to value “typical” views of peer friendships rather than personally meaningful social connections (Lisboa White et al., 2024). Second, ill-being has been operationalized using tools that are normed with the general population; thus, their validity for assessing autistic adults’ mental health is questionable. For example, the *Hamilton Depression Rating Scale* and the *Beck Depression Inventory* might misrepresent meaningful autistic strategies such as a stimming or engaging in interests as markers of depression (Stewart et al., 2006). Third, ways in which autistic people can experience emotional distress may be missed in current understandings of mood disorders. Milder emotions may go unnoticed until they accumulate into an experience of overload, and the fatiguing impact of experiencing sensory strain likely goes unrecognized as part of emotional wellbeing (Petty et al., 2023b; Smith & Sharp, 2013). Moreover, an estimated 33%–63% of autistic people are thought to experience alexithymia (Kinnaird et al., 2018), defined as difficulties in distinguishing or describing your emotions (Bird & Cook, 2013), which general assessment tools are not sensitive to (Curnow et al., 2023). The development of appropriate therapeutic supports for autistic adults depends on the ways in which autistic wellbeing is conceptualized and measured, and on the resultant research that informs clinical developments (NICE, 2012b). Psychological interventions are incomplete if they are not informed by first-person perspectives on subjective wellbeing. For autism, this means understanding how wellbeing interacts with characteristics of autistic phenomenology, intersectional complexities, and support needs (Deakin et al., 2024; Pantazakos & Vanaken, 2023).

Appropriate sampling remains an issue in autism research (D'Mello et al., 2022; Girolamo et al., 2023). Autism is formally diagnosed based on assessments of communication, social, sensory, and behavioral presentations (APA, 2013). However, these offer brief and static descriptions that mean many autistic people do not see themselves represented in diagnostic criteria (Kapp & Ne’eman, 2020). The assumption that autistic people are a homogenous group is also problematic (Heasman & Parfitt, 2023). Underlying these problems is the diagnostic process itself. Diagnostic decisions are substantially influenced by qualitative impressions from brief interactions in diagnostic spaces (Hobson & Petty, 2021). Assessment pathways likely exclude people who face barriers to access, who are on waiting lists, or who are not well represented by the latest definitions of being autistic, including younger females and people from minoritized ethnic backgrounds (Aylward et al., 2021; Lockwood Estrin et al., 2021). There are also problems in both the availability of suitably designed research studies and access to participation in research for autistic people (Russell et al., 2019), highlighting the need for participatory approaches. Sampling, therefore, determines who is fairly represented in research conducted about autistic people. A second associated consideration for sampling is whether autistic people are accurately represented by ratings of “autistic traits.” Measurement of “traits” assumes that participants with higher scores will mirror the behavioral profile of people clinically diagnosed as autistic (English et al., 2021). However, brief psychometric tools are not a comprehensive clinical assessment and may place disproportionate weight on particular characteristics (Ashwood et al., 2016). Given these issues, this review critically appraises the sampling designs in research on autistic wellbeing (D'Mello et al., 2022).

Importantly, a new ontological understanding of autism has provided the opportunity to refocus research towards understanding autistic phenomenology (Pantazakos, 2023; Pellicano & den Houting, 2022). For example, the articulation of the double empathy problem has changed the conceptualization of social communication difficulties in autism (Milton, 2012; Milton et al., 2022). From this perspective, social communication breakdown is understood as being intersubjective and experienced mutually by both autistic and nonautistic people, rather than the direct result of an intrinsic autistic “deficit” (Heasman & Gillespie, 2018). The relatively new concept of “autistic burnout” is a second example of how issues of wellbeing have been reframed as interactional, such that cumulative normative expectations placed on an autistic person might negatively impact their wellbeing (Raymaker et al., 2020). These conceptual developments have often been led by autistic scholars and activists, showing that centring first-person perspectives can lead research into more valued directions for autistic people (Pukki et al., 2022). Importantly, this ontological reframing has led to therapists being asked within practice to develop a reasonable understanding of how autism and mental ill health co-occurrences are separable (Chapman & Botha, 2023; Petty et al., 2023a). The extent to which research into psychological interventions for autistic people has incorporated these conceptual shifts is unknown.

The current study aims to advance understanding of how “improved wellbeing” for autistic people is conceptualized within intervention research literature. The National Institute for Health and Care Excellence (NICE) provide guidance and best practice support to the UK's National Health Service (NHS). However, NICE's ability to make recommendations for best clinical practice for wellbeing interventions for autistic adults is limited by a lack of quality evidence (NICE, 2012b). In the absence of robust research, NICE GC142 recommends that existing interventions for a specific mental health condition (e.g., anxiety or depression) should be used with autistic adults with delivery adaptations, which leaves much room for further direction. Previous systematic reviews or meta-analyses have evaluated either single interventions, for example, cognitive behavior therapy (CBT) (Weston et al., 2016), single outcomes, for example, social anxiety (Spain et al., 2018), or a single study design, for example, randomized control trials (Linden et al., 2023). These reviews collectively conclude that the ability to make robust recommendations is limited until more definitive trials are undertaken. Importantly, these reviews do not interrogate whether the interventions being evaluated are appropriately designed for autistic people. In contrast, the current review does not seek to evaluate the efficacy of interventions, but rather builds a picture of the breadth of psychological intervention approaches being taken, the rationale adopted for how wellbeing can be improved, and how psychological interventions are designed and evaluated for autistic adults specifically. The research objectives were to identify: (1) The extent to which measures used to evaluate psychological interventions have been designed or confirmed to be suitable for use with autistic people; (2) the extent to which elements to change or reduce core autistic presentations have been included in psychological interventions used with autistic adults; (3) the candidate mechanisms for change, hence the suggested reasons for improved wellbeing; and (4) the adaptations made to existing psychological interventions for autistic adults.

## Method

A protocol was registered with PROSPERO before commencement (reference CRD42023385232). The review was reported according to Preferred Reporting Items for Systematic Reviews and Meta-Analyses (PRISMA) guidelines for conducting systematic reviews (Page et al., 2021).

### Review Criteria

PRISMA 2020's Population, Intervention, Comparator, Outcome (PICO) framework was used to define the criteria for the review (Table 1). To develop the search strategy, a Patient and Public Involvement (PPI) process sought input from an expert panel of autistic counselors who work with autistic clients (NHS Health Research Authority, 2020). The counselors volunteered to inform the study design after responding to an invitation to professional networks.

**Table 1.** PICO and inclusion/exclusion criteria.

Study Criteria	Inclusion	Exclusion
Design	Any empirical study design, including *n* = 1 case studies. Qualitative, quantitative and mixed method studies. Primary intervention studies or follow-up designs, with at least one pre and post psychological wellbeing outcome measure completed by a clinician, a participant, or other party such as a carer or family member.	Reports, commentaries, letters, editorials, other nonprimary studies. Multiple papers for the same study. Studies where no psychological wellbeing measure had been used, for example, only biological or physiological measures were used.
Date	Studies published between 2012 and 2023.	Studies published prior to 2012.
Language	Studies published in English.	Non-English language studies.
Participants	Adult autistic participants without intellectual disability. Where autistic participants within studies were described by one of the terms: Autistic, with Autism, Asperger's syndrome, Pervasive Development Disorders, or their abbreviations (ASC, ASD, PDD). The descriptor “High Functioning Autism” (HFA) was included as research studies may have made use of it (Kenny et al., 2016). Studies must have verified participants autism diagnosis or assessed levels of autistic “traits.” Studies must include at least one adult participant, aged 18 over.	Studies designed for autistic participants with an intellectual disability. Studies with self-identified autistic participants where no form of study verification of diagnosis or autistic “traits” was undertaken. Studies where it was not possible to determine whether adults (aged 18 or over) were included.
Interventions *Syntax adapted to cover English/US spelling and different word endings, to capture:*	Interventions delivered in any format, including self-help, individual, couples or group based. There were no restrictions on the delivery of the intervention, which could include talking therapies, relaxation or activity based. Dialectical behaviour therapy (DBT) Cognitive remediation therapy (CRT) Eye movement desensitization or EMDR Mindfulness (MBSR) Mentalization Cognitive behaviour therapy (CBT) Behaviour activation or behaviour therapy Interpersonal therapy (IPT) Acceptance and commitment therapy (ACT) Counselling Person centered therapy Behaviour therapy Applied behaviour analysis Independent living, practical or peer support.	Where the intervention was not for one of the outcomes listed below.
Comparisons	With or without a comparator participant group.	Not applicable.
Outcomes *Syntax adapted to cover English/US spelling and different word endings, to capture:*	Measures of the following representations of wellbeing: Depression or low mood Obsessive compulsive disorder (OCD) Anxiety, panic Stress Loneliness or isolation Identity Self-worth Sleep Anger Phobia, hypochondria Anorexia, eating, food related behaviours Alcohol, substance use Schizophrenia disorder Agoraphobia, dysmorphic Trauma and Post-traumatic stress disorder (PTSD) Self-harm, suicidality Emotion regulation Quality of life, wellbeing Autistic burnout.	Where the intervention was not one of those listed above. Where the primary aim of the intervention was to: deliver social skills training programes OR promote camouflaging of autistic behaviours.

PICO = population, intervention, comparator, outcome; EMDR = eye movement desensitization and reprocessing.

### Study Design

All study designs were included in the review in order to meet the objective of summarizing all available evidence since 2012, the point at which NICE introduced Clinical Guidelines for working with autistic adults (NICE, 2012a).

### Participants

The review was restricted to studies where participants did not have an intellectual disability (ID). Autistic people represent heterogeneous characteristics, co-occurrences, and intersectionalities (Aylward et al., 2021; Lai et al., 2019), covering additional factors such as co-occurring ID may have impacted the sensitivity of the review. Improving understanding of effective wellbeing interventions for autistic people with ID is important, despite not being the focus of this review.

The search strategy included the descriptor “High Functioning Autism” (see Table 1), because it has been used in the research despite being criticized as a unidimensional and misleading functional label (Kenny et al., 2016).

### Interventions

A list of psychological interventions was identified through the PPI process. No restrictions were applied to the delivery of the intervention, which could include talking therapies, relaxation, activity-based methods, and any format including self-help, individual, or group-based formats. Studies that measured physiological attributes (e.g., heart rate or cortisol levels) or used neuroimaging outputs were included if they also used psychological wellbeing outcome measures.

### Outcomes

Wellbeing was represented by a broad list of search terms to provide a comprehensive view of the available research (Petticrew & Roberts, 2006). In addition to wellbeing and quality of life (QoL) indicators, outcome measures encompassed ratings of mental health and psychiatric conditions (including anxiety and Obsessive Compulsive Disorder [OCD]) and emotional difficulties (including anger and emotion dysregulation). This breadth of outcome measurement reflected the available literature; we acknowledge that wellbeing is more than the absence of mental ill health (Dodge et al., 2012). However, there is currently no stable definition of wellbeing for autistic people (Deakin et al., 2024; Lam et al., 2021). We advocate a conceptualization grounded in the experiences of autistic people, rather than “adding to” wellbeing concepts developed for the general population. Some terms historically used within autism research are now considered to be exclusionary and potentially inaccurate (Dwyer, 2022; Kenny et al., 2016). Whilst we favor using value-neutral descriptive language (Chapman, 2020a), the search strategy was developed to ensure a comprehensive review.

The outcomes list was derived from the reporting categories within UK statistics of psychological therapy (NHS Digital, 2022) and then expanded via the PPI process.

### Search Strategy

A search strategy was developed (including the appropriate syntax and natural language terms) with expertise from a subject-specific librarian and extensive scoping exercises. Subject headings (CINAHL/MeSH) were unsuccessful, returning too few studies when compared with natural language search terms alone. Protocol-driven searches were conducted in four bibliographic databases: APA PsycInfo, APA PsycArticles, MEDLINE and CINAHL until January 2023. These searched for English Language studies published since 01 January 2012. The Boolean search strings used are in Online Resource 1. Searches were performed on the gray literature database OPENGREY.EU until January 2023, but no records were identified. Extensive hand searching was undertaken, as recommended for heterogeneous reviews (Greenhalgh & Peacock, 2005). A “Gold List” of studies of potential interest was built from previous systematic reviews, and forward-and-backwards citation searching was conducted for studies identified for inclusion via the protocol-driven search.

### Selection Process

**Figure 1.**
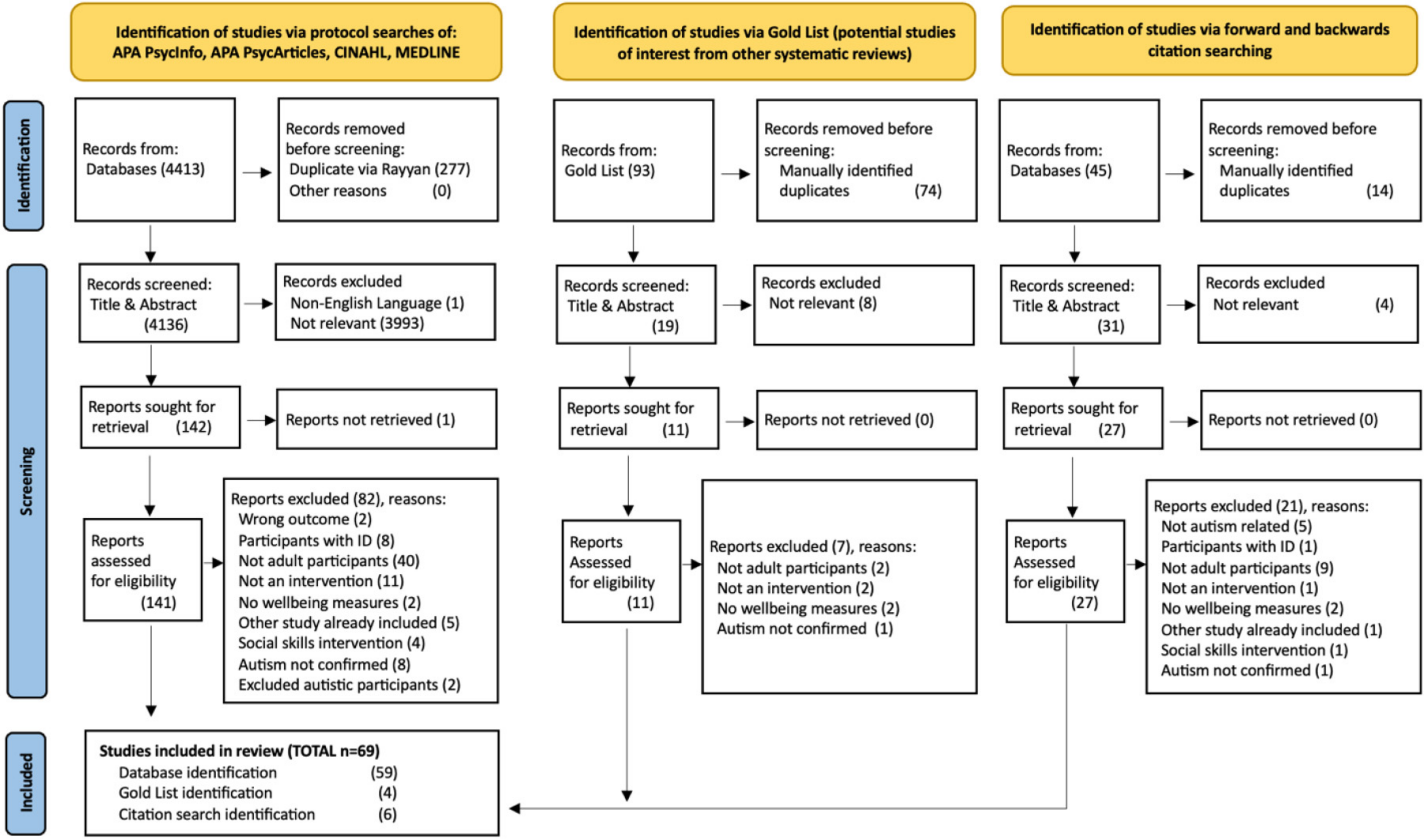
PRISMA 2020 flow diagram for the systematic protocol and hand searching of relevant records.

Screening was performed on 100% of the records identified by the protocol-driven search (Figure 1) by two independent reviewers: The first author and a research assistant. Blind reviewer functions within Rayyan software were used to ensure independent checking. Differences were resolved in team discussions and mostly related to whether an outcome measure captured an aspect of wellbeing. Forward-and-backwards citation searching and searching for citations within other systematic reviews (gold list) were undertaken by one reviewer.

### Assessment of Quality and Risk of Bias

Two established tools were used. First, the 16-item Quality Assessment Tool for Studies with Diverse Designs (*QATSDD*) (Sirriyeh et al., 2012). Questions 3, 7, 8, and 9 were omitted as they replicated areas investigated via the second assessment tool. Items are scored from 0 to 3, with total scores converted to a percentage. Studies were assessed by one reviewer, and a randomly selected 20% sample of papers was reviewed independently by a second reviewer. Initial discrepancies in scores were addressed through a joint review of higher-quality studies as a calibration exercise. Second, the risk of bias (RoB) tool (Table 2) was based on a rating chart used in previous autism studies (Stewart et al., 2022; Wigham et al., 2017). Modifications broadened the scope of the questions originally applied to depression measures and alignments with current language preferences (Dwyer, 2022). The “medium risk” category was re-labelled as “some concerns.” Question 6 of the original tool assessed the risk for the assessment of participants’ intellectual ability. This question was omitted from this review as some measures of intelligence may be less reliable in measuring IQ with autistic people (Dawson et al., 2007; Nader et al., 2016). Therefore, their use may not support a robust assessment of the RoB. Bias assessment values were based on the information provided within the original studies, using the authors’ statements of whether outcome measures were considered reliable to use with either the general population or autistic participants. The study level RoB for the fourth and fifth risks (assessing the stated validation of outcome measures) was calculated so that (a) if each measure was assessed at the same RoB this became the study's RoB level, or (b) if assessments of bias were a mix of risk levels, then a study level RoB of some concerns was assigned, regardless of the number of measures at each risk level, or (c) where one of the measures or submeasures was not identified as having validity with autistic adults.

**Table 2.** Risk of bias rating chart adapted from Wigham et al. (2017)

	Risk of Bias	Description
1	**Verification of participant's autism diagnosis**	
	Low risk	A diagnosis of autism was confirmed at the time of the study by standardized test, undertaken by a psychologist, psychiatrist or reliable expert in using DSM or ICD diagnostic criteria OR diagnosis was confirmed by a comprehensive validation of medical records OR participants were recruited via a route that confirmed participants were autistic.
	Some concerns	A diagnosis of autism was made as part of study inclusion procedures. It was either not stated who performed this, OR they were part of the research team and not an expert in undertaking autism diagnostic assessments OR the study stated they confirmed the diagnosis according to evidence, but there was a lack of detail about the process OR participants were recruited via a route that confirmed participants were autistic but there were insufficient details about the source.
	High risk	Either a self-report or researcher-completed questionnaire of autistic “traits” was undertaken with no further verification.
2	**Description of participants**	
	Low risk	Key characteristics described including mean age, age range, gender, co-occurring conditions and medications.
	Some concerns	Descriptive characteristics were included but some details were missing, for example ethnicities and other co-occurrences OR reference was made to where further details could be found.
	High risk	Key demographic information was missing.
3	**Description of recruitment**	
	Low risk	Recruitment process was described including geographical area, method of referral (e.g., self, database), and setting (e.g., clinic or school).
	Some concerns	Information is provided but misses details.
	High risk	Recruitment process was not described.
4	**Validity and standardization of outcome measure for the general population**	
	Low risk	Standardized measure or sub-measure identified as having validity with the general population.
	Some concerns	Measure used previously within research studies but there was a lack of clarity about its validity status with the general population.
	High risk	Nonstandardized measure or psychometric properties were not reported for its use with the general population.
	NA (not applicable)	Reliability and validity of the outcome measure's use for the general population was not applicable as the study exclusively included participants considered to be autistic.
5	**Validity and standardization of outcome measure for autistic people**	
	Low risk	Standardized measure or sub-measure identified as having validity with autistic people.
	Some concerns	Measure used previously in research studies but there was a lack of clarity about its validity status with autistic people.
	High risk	Nonstandardized measure or psychometric properties were not reported for its use with autistic people.

### Synthesis Methods

Meta-analysis was inappropriate due to the expected heterogeneity of study designs, interventions, and outcomes (Higgins & Green, 2008). Mixed-methods and qualitative synthesis methods were used, including textual narrative syntheses and mixed-methods synthesis matrices (Barnett-Page & Thomas, 2009; Booth et al., 2016; Popay et al., 2006). Textual narrative syntheses were undertaken separately for 42 studies with adult participants (AO) as one reporting category, and 27 studies with mixed adult and child participants (AC). This was so that a robust picture of research across the lifespan with autistic adults was presented independently of research with children.

A framework synthesis approach was used for the interpretative analysis of AO studies. Three framework syntheses (Barnett-Page & Thomas, 2009) had been identified a priori for the interpretive synthesis of studies with adult participants: Adaptations made for autistic adults to therapeutic protocols and their perceived contribution to outcomes; underlying candidate mechanisms of change for autistic people; and reasons and methods for the inclusion of social skills training within wellbeing interventions. An additional framework was added based on its relevance to the integrative analysis of the use of outcome measures (Booth et al., 2016): Participants’ views of the use of self-report outcome measures. Data were extracted from qualitative results and discussion sections, and then synthesized with continuous referencing back to the original papers. The terminology used within and across the studies was variable, and therefore, a full line-by-line review was undertaken. Data were sought deductively relating to: Participants’ views of the use of self-report outcome measures; and social skills training as a component within intervention protocols; and inductively for the: Identification of candidate mechanisms; and adaptations made within protocols for autistic participants.

## Results

Results are presented as: Study selection results; descriptions of studies; study quality; RoB results and Research Objective 1; then results addressing each of the remaining 3 research objectives.

## Study Selection Results

The search of electronic databases retrieved 4,413 records that were reduced to 4,136 after de-duplication. Handsearching identified a further 50 papers, 40 of which were subsequently excluded during full screening (Figure 1).

In total, 4186 records were screened by two independent reviewers: Screening of TITLE and ABSTRACT excluded 4,006 records; 180 records went forward for full text review, at which point 111 papers were excluded. Disagreements were resolved by joint discussion, and exclusion reasons recorded. Cohen's kappa coefficient for inter-rater reliability (*K* = 0.86) indicated almost perfect agreement over which studies to include at full-text review. The final dataset comprised 69 papers.

## Description of Studies

Studies used a range of study designs, participant age ranges, interventions, and outcomes. Countries contributing more than one study were: USA (*n* = 20), UK (*n* = 13), the Netherlands (*n* = 10), Sweden (*n* = 8), Japan (*n* = 7), and Australia (*n* = 3). Countries contributing one study were: Canada, Ireland, Korea, France, Switzerland, Norway, and Israel. One study was internationally based at locations in Canada, Denmark and the Netherlands (Ridderinkhof et al., 2021).

Summary rather than specific descriptions were often used to identify study design (e.g., “pilot study”), and six studies did not state a design, indicating a lack of methodological detail. Quasi-experimental (QE) pre/post-test designs with no comparison groups were the most common design (*n* = 30), reflecting the exploratory nature of studies. Other designs were RCTs (*n* = 19); QE multi-condition studies (*n* = 11); and case-based designs (*n* = 9).

Sample sizes were typically small. Forty-nine studies had sample sizes of less than 40 participants. Based on this review, few autistic adult participants have been involved in psychological intervention research for improved wellbeing since 2012. The 42 AO studies included 1267 autistic adults, 34 of whom were classed as autistic based on rating scales of autistic “traits” rather than a confirmed diagnosis. A consistently low number of empirical studies were published during the 11 years covered by the review (one or two per year). The number of pilot studies published annually increased from 2019, with 13 published in 2022. Twelve studies were larger-scale evaluations of effectiveness with 50 or more participants.

The mean age and age ranges of participants were inconsistently reported (Online Resource 2). The mean participant age across AO studies was 32.7 years (SD 6.99). Studies tended not to consider differences between birth sex and gender orientation; most reported participants as male or female. There was a higher number of male participants within the studies (*n* = 785) than females (*n* = 441) or other identified genders (*n* = 41). A Chi-Square Goodness of Fit Test with an assumed equal proportion of male/female participants identified that, for 38 of the adult participant studies, males were significantly more likely than females to be included as participants, *X^2^*(1, *N* = 1,198) = 83.35, *p*<.001. Four studies did not report gender or used exclusive sample strategies.

It is not possible to know how many adult autistic participants were represented within the 27 studies that had a mix of adult and child participants due to limited sample descriptions and the use of average ages. Therefore, only major points from the synthesis are reported (Online Resource 3). The 27 studies focused on: (1) Children up to 18 years old (*n* = 4); (2) adolescents, defined here as participants aged between 12 and 25 years (*n* = 14); and (3) studies with a broad participant age range (*n* = 9). Ekman and Hiltunen (2015) reported separately on teenage participants and adult participants. The remaining eight did not provide theoretical arguments for how the intervention would be appropriate for both children and adults. Example age ranges were: 10–35 years (Zaharia et al., 2021) and 7–24 years (Jackson et al., 2022).

## Study Quality

Quality scores produced using the *QATSDD* (Sirriyeh et al., 2012) ranged from very poor to excellent, but 38 of the 69 studies scored less than 50%, indicating methodological limitations in the majority of studies. All studies were retained as potentially informative in answering the research questions. However, the absence and variability of reported information impacted some syntheses. For example, the inconsistent recording of participant characteristics meant that attrition rates were not compared.

## Risk of Bias

Assessments were based on published information; study authors were not contacted for further clarification that may have been available (Online Resource 4 has the full synthesis). A category of “not applicable” indicated the RoB had no relevance to that study. Table 3 summarizes RoB totals in two categories: Studies with only adult participants (AO), and studies that included child participants alongside adult participants (AC).

**Table 3.** Number of studies for each risk of bias (RoB) level within five domains.

		Domains				
		Verification of Participants	Description of Participants	Description of Recruitment	Validity and Standardization of Outcome Measure for	
Study Categories	RoB Level	Autism Diagnosis			General Population	Autistic People
Studies with adult participants only (AO)	Low risk	29	9	27	2	0
	Some concerns	11	30	13	1	15
	High risk	2	3	2	2	27
	Not applicable				37	
**AO total**		**42**	**42**	**42**	**42**	**42**
Studies with adult and child participants (AC)	Low risk	16	4	24	2	0
	Some concerns	11	23	3		14**
	High risk					13
	Not applicable				25	
**AC total**		**27**	**27**	**27**	**27**	**27**

**Three studies used all validated measures, validated with children or adolescents.

### Verification of Participants’ Autism Diagnosis

Verification methods for confirming participants’ autism diagnoses varied; 45 studies had low RoB (a positive marker) through undertaking comprehensive validation of existing medical records or appropriate diagnostic assessments. Most studies (*n* = 30) used the *Autism Diagnostic Observation Schedule* (ADOS, ADOS-2 or ADOS Module 4), and 17 studies used the *Autism Diagnostic Interview* (ADI or ADI-R). The ADOS-2 and ADI-R are viewed as gold standard diagnostic tools to inform autism assessment, though they are used as one aspect of formal diagnosis (NICE, 2021), and were sometimes used interchangeably in the same study, for example, Spain et al. (2017). Studies received a RoB of some concerns (*n* = 22) for a lack of detail or if the appropriateness of the assessor was unknown. Two studies resulted in a high RoB for participants’ autism diagnosis as no assessment beyond recording total screening scores (via the ADI-R, ADOS-2, or *Autism Spectrum Quotient* (AQ)) was undertaken.

Some studies additionally measured participants’ autistic “traits,” most commonly with the AQ or a short-form variant. Reasons were to describe participants’ clinical characteristics at baseline (*n* = 15) or to report outcomes in groupings associated with score levels (*n* = 4). A further three studies assessed whether autistic “trait” scores changed (de Bruin et al., 2015; Hesselmark et al., 2014; Ishii et al., 2022), demonstrating that some studies evaluating psychological interventions expected autistic characteristics to change.

### Description of Participants and Recruitment

The majority (*n* = 53) of studies were rated as having “some concerns” with the reporting of participant characteristics, as participants’ co-occurring conditions, medication, and/or ethnicities were not reported. This mirrors previous research (Cook et al., 2021; Girolamo et al., 2023), impacting the ability to understand whether the full complexities of each participant's life context or wellbeing were represented. Reporting participants’ co-occurring neurodivergences was rare (Lobregt-van Buuren et al., 2019; Pahnke et al. 2014, 2019 and 2022; Quadt et al., 2021;).

Participants were recruited via three main routes: Social media, autism charities, and mental health services. Settings included clinics, inpatient wards, and school/university campuses. Most studies (*n* = 51) had low RoB relating to recruitment details as they provided clear descriptions of the setting and processes.

## Objective 1: The Extent to Which Measures Used to Evaluate Psychological Interventions Have Been Designed or Confirmed to be Suitable for use with Autistic People

### RoB Validity and Standardization of Outcome Measures for Autistic Participants

Research Objective 1 for this review was whether intervention studies used wellbeing measures that were known to be validated with autistic adults. Bias assessment values are based on the information provided within the original study documentation for clinician, parent/caregiver reports, and self-report measures used in initial assessments, interventions, or postintervention. Measures used to confirm autism as part of the inclusion process, economic evaluation, and parent/carer measures were excluded. Also excluded were physiological attributes (heart rate or cortisol levels) or neuroimaging measures.

The majority (*n* = 40) of studies were rated as high RoB. Authors either (a) acknowledged that their measures were not standardized for use with autistic people, or more commonly, (b) made no comment on their status for use with autistic people. Twenty-nine studies were categorized as having some concerns. In 26 of these, authors combined measures validated with autistic people with measures previously used in autism research. Three studies exclusively used validated measures, but these were validated with autistic children rather than adults. Murray et al. (2015) used the *Children's Yale-Brown Obsessive–Compulsive Scale*, Carey et al. (2022) used the *Anxiety Scale for Children for ASD,* and Murphy et al. (2017) used three measures validated with autistic children or adolescents. The RoB figures for validity and standardization of outcome measures for autistic participants suggest either that study authors did not consider the standardization of outcome measures with autistic people, or that they struggled to identify appropriately validated measures.

A total of 257 outcome measures or sub-measures were named in the studies, of which 35 (14%) were identified by study authors as having validity for use with autistic people (Table 4). Examples include a self-report anxiety questionnaire that has been developed collaboratively with, and validated for autistic children, the *Anxiety Scale for Children for ASD* (Carey et al., 2022)*.* An adult version has since been developed, *Anxiety Scale for Autism-Adults* (Rodgers et al., 2020), but was not used in studies in this review.

**Table 4.** Outcome measures and subscales reported as validated with autistic people.

No	Outcome Measure Or Subscale
	**E** **motional distress and co-occurring characteristics**
1	ADIS-C/P—Anxiety Disorders Interview Schedule for Children/Parents
2	ASC-ASD—Anxiety Scale for Children for ASD
3	BDI-II—Beck Depression Inventory, Second Edition
4	BYI—Beck Youth Inventories, subscales for anxiety, depression and anger.
5	CASI-anx—Child and Adolescent Symptom Inventory-4 ASD Anxiety Scale
6	CY-BOC—Children's Yale-Brown Obsessive–Compulsive Scale
7	DASS-21—Depression Anxiety Stress Scales
8	DERS—Difficulties in Emotion Regulation
9	FFS—Flinders Fatigue Scale
10	GAFS-8—General Alexithymia Factor Score
11	HADS—Hospital Anxiety and Depression Scale
12	OCI-R—Obsessive Compulsive Inventory–Revised
13	SSS—Stress Survey Schedule for Persons with Autism and Other Developmental Disabilities
14	Stress—Survey Schedule for Autism and Other Developmental Disorders
	**Sleep quality**
15	PSQI—Pittsburgh Sleep Quality Index
16	SAAQ—Sleep Anticipatory Anxiety Questionnaire
	**Behaviour characteristics**
17	BRIEF-2—Behavior Rating Inventory of Executive Function, second edition
18	CSBQ—Children's Social Behavior Questionnaire
19	SCQ—Social Communication Questionnaire—Lifetime Version
20	SIAS—Social Interaction Anxiety Scale
21	SPS—Social Phobia Scale
22	SRS—Social Responsiveness Scale
23	SRS-A—Social Responsiveness Scale, Autism
24	SRS—Social Responsiveness Scale, Dutch version
25	SRS-2—Social Responsiveness Scale-2—Adult Self-Report
	**Intelligence**
26	KWAIS-IV Korean-WAIS
27	WASI-II—Wechsler Abbreviated Scale of Intelligence—Second Edition
28	WASI—Wechsler Abbreviated Scales of Intelligence
	**Mindfulness ability**
29	CAMM—Child and Adolescent Mindfulness Measure
	**Perceived QoL**
30	SWLS—Satisfaction with Life, Global QOL satisfaction and wellbeing
31	WHOQOL-DIS-ID WHO Disability
32	WHOQOL-BREF-ID World Health Organization (WHO) QoL
33	WHOQOL-BREF Abbreviated WHO QOL Questionnaire
	**Expectations of treatment**
34	TCS—Treatment Credibility Scale
35	TSC ASD adapted version of the Treatment Credibility Scale

ID: intellectual disability; QoL: quality of life.

### Qualitative Feedback About the use of Self-Report Questionnaires (AO Studies)

This textual narrative synthesis adds to the RoB synthesis on the extent to which outcome measures had been standardized for use with autistic adults. Multiple issues were reported relating to the use of outcome measures. A nested qualitative investigation with 21 participants captured many of these (Russell, Gaunt et al., 2019). For example, three participants found the self-report questionnaires helpful in highlighting positive changes, and conversely, one participant was discouraged by having their difficulties highlighted by questionnaires. Some participants reported that filling in questionnaires was time-consuming and unhelpful. They reported struggling to quantify their experiences on the scales used: For instance, finding it difficult to put a number to the frequency of their symptoms. Participants in Russell, Gaunt et al., 2019 study also reported that many questions appeared to ask about similar concepts or were vaguely expressed, which could be confusing and elicit quick and unconsidered answers. Similar experiences were reported in other studies (Gaigg et al., 2020; Hesselmark et al., 2014; Lobregt-van Buuren et al., 2019). In two studies, findings could either indicate genuine null results or insufficiently sensitive outcome measures for autistic people. First, contrary to expectation, the *Mindfulness Attention Awareness Scale* outcomes did not change during the intervention period and at follow-up (Sizoo & Kuiper, 2017). Second, a lack of change in scores postintervention in the *Work and Social Functioning* general measure of functioning led authors to suggest that the measure may not be sufficiently sensitive to assess change (Spain et al., 2017). A small subset of studies included qualitative descriptions of participants’ perspectives on outcome measures, thus limiting the ability to draw meaningful conclusions. Yet these preliminary observations are important because they suggest that some autistic people do not see the relevance to their experience of some outcome measures and/or find them imprecise and difficult to complete accurately. Further qualitative exploration of autistic adults’ experiences of completing questionnaires for outcome measures is recommended.

## Objective 2: The Extent to Which Elements to Change or Reduce Core Autistic Presentations Have Been Included in Psychological Interventions Used with Autistic Adults

This section presents: Analysis of outcomes, and the interpretive syntheses of the use of social skills training in the 42 AO studies, followed by an analysis of outcomes in the 27 AC studies.

### Outcomes (AO Studies)

A total of 17 wellbeing outcomes were represented. Outcomes were commonly not identified as being primary or secondary (See Online Resource 3). Specific outcomes identified in more than one study were: Anxiety (*n* = 12); depression (*n* = 9); QoL/wellbeing (*n* = 8); emotion regulation skills (*n* = 5); stress (*n* = 4); social functioning/skills/communication (*n* = 4); OCD (*n* = 4); social anxiety (*n* = 3); sleep disturbances (*n* = 3); cognitive flexibility/functioning (*n* = 3); and substance abuse (*n* = 2). Self-harm and suicide ideation, trauma, specific phobia, distressing thoughts, autistic “symptoms,” and autism awareness were outcomes in one study each.

A third of the studies (*n* = 14) stated objectives to contribute to improvements in several, outcome measures (Bemmouna et al., 2022; Brezis et al., 2021; Hare et al., 2016; Hartmann et al., 2019; Hidalgo et al., 2022; Kiep et al., 2015; Okuda et al., 2017; Ordaz et al., 2018; Oshima et al., 2021; Oswald et al., 2018; Pahnke et al., 2019; Pahnke et al., 2022; Ritschel et al., 2021; Spain et al., 2017). Objectives represented: (1) Reducing specific aspects of ill-being, for example, symptoms of anxiety and depression; (2) generalized improvements in multiple domains, for example, QoL, stress; (3) increasing emotion regulation or cognitive functioning skills; or (4) increasing social functioning skills (as based on rating scales).

**Figure 2.**
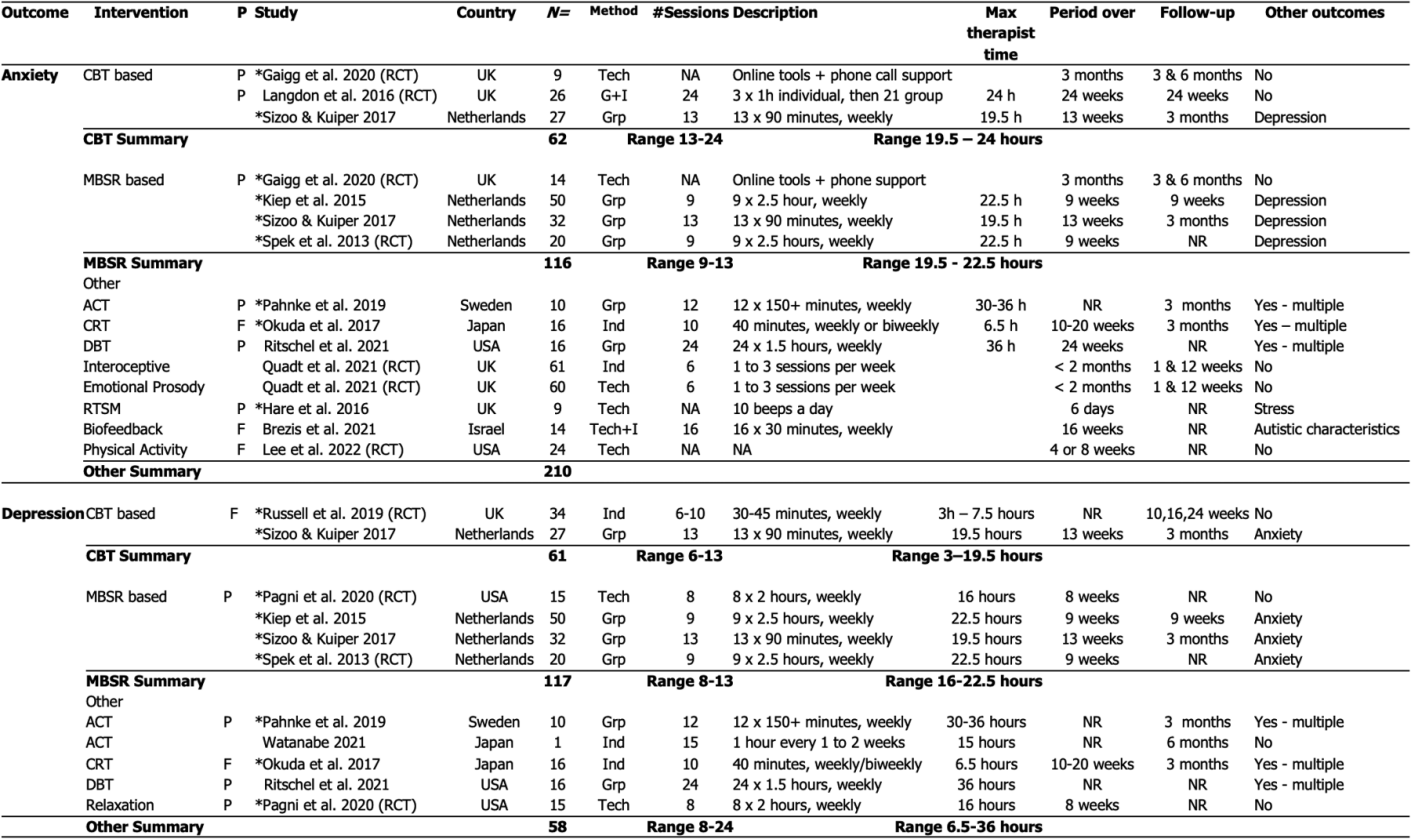
Comparison of intervention characteristics for studies with adult participants (AO) where anxiety or depression was an outcome.

A separate synthesis compared intervention protocols where anxiety or depression were outcomes (Figure 2). Studies to reduce symptoms of anxiety included 388 autistic adults; these were predominantly pilot designs. Studies focused on reducing symptoms of depression had 236 autistic participants. This synthesis demonstrates the low number and small scale of clinical research studies for autistic adults with reported co-occurrences of anxiety or depression.

### Social Skills Training as a Component Within Intervention Protocols (AO Studies)

This review purposely excluded interventions where the *primary* outcome was the acquisition of social skills, but retained studies where social skills were included as an outcome used alongside wellbeing measures. Social skills training is a psycho-educational approach to building knowledge of interpersonal relations (NICE, 2021). Social skills training can be contentious if based on the assumption that there is a “correct” (neuronormative) way to interact socially that autistic people should adopt (Bottema-Beutel et al., 2018; Milton & Moon, 2012). However, social skills training is a recommended approach in the United Kingdom for autistic adults to develop self-advocacy and adaptive functioning skills (NICE, 2021).

Specific reference to improved social functioning skills was made in seven studies (Bemmouna et al., 2022; Hartmann et al., 2019; Hesselmark et al., 2014; Hidalgo et al., 2022; Langdon et al., 2016; Oswald et al., 2018; Spain et al., 2017). Social skills training was included for multiple reasons. These were: To support participants in developing greater interpersonal effectiveness; to build adaptive functioning skills (e.g., making telephone calls or asking for help); and to increase knowledge about dominant social norms to reduce feelings of anxiety relating to social interactions. Methods used were predominantly didactic lectures, modeling of behaviors, and in vivo practice. Improved social functioning was reported in three studies (Hartmann et al., 2019; Oswald et al., 2018; Spain et al., 2017). Other outcomes included improving QoL and reducing symptoms of anxiety, emotion dysregulation, or stress. There was no explicit detail provided in the protocols in two studies (Hesselmark et al., 2014; Langdon et al., 2016). Increased inclusion of social skills elements was requested by participants in two studies (Langdon et al., 2016; Oswald et al., 2018).

### Outcomes (AC Studies)

As with the adult-only participant studies, there was often no primary outcome specified: Reducing symptoms of anxiety was the most common outcome (*n* = 12). Studies included outcomes described as a reduction in autistic “symptoms” (de Bruin et al., 2015; Ehrenreich-May et al., 2020; Salem-Guirgis et al., 2019; Yang & Chung, 2022), “behaviour” (Jackson et al., 2022), or “excessive or avoidance behaviours” (Ekman & Hiltunen, 2015), and an increase in “social functioning” (Bemmer et al., 2021; Connor et al., 2020; Jackson et al., 2022; Ridderinkhof et al., 2018). These were summary descriptions where studies assessed participants’ behavioral and social characteristics via rating scales, for example, the *Behavior Assessment System for Children* (Jackson et al., 2022; Salem-Guirgis et al., 2019), and the *Aberrant Behavior Checklist,* a caregiver assessment rating characteristics described as social withdrawal, crying, inappropriate speech, and behavior (Yang & Chung, 2022). Data for Research Objective 2 identified that a minority (*n* = 7) of studies reviewed followed a medical model conceptualization of autistic characteristics (i.e., had outcomes to reduce core autistic presentations or change behaviors) equating improved well-being to changing autistic “traits” or behaviors (Brezis et al., 2021; de Bruin et al., 2015; Ehrenreich-May et al., 2020; Ekman & Hiltunen, 2015; Jackson et al., 2022; Salem-Guirgis et al., 2019; Yang & Chung, 2022). Six of these were AC studies. A further minority (*n* = 7) AO studies included social skills training to increase adaptive functioning skills, there was insufficient detail to know whether training encouraged altering core autistic characteristics.

## Objective 3: Identification of Candidate Mechanisms for Change (AO Studies)

Candidate mechanisms of change are defined broadly here as any process or event considered as a potential reason for change in wellbeing for autistic people following psychological intervention (Kazdin, 2007). Five candidate mechanisms were proposed by the study authors (Online Resource 5): (1) Improved emotion regulation skills in 11 studies (Beck et al., 2020; Bemmouna et al., 2022; Brezis et al., 2021; Hare et al., 2016; Kuroda et al., 2022; Lawson et al., 2022; Pahnke et al., 2019; Pahnke et al., 2022; Quadt et al., 2021; Sizoo & Kuiper, 2017; Tchanturia et al., 2016); (2) reduced rumination contributing to reducing symptoms of anxiety and depression (Kiep et al., 2015; Spek et al., 2013); (3) increased cognitive defusion skills to reduce the impact of emotional distress in the moment (Maisel et al., 2019); (4) reduced insomnia symptoms contributing to reduced symptoms of anxiety and depression (Ishii et al., 2022; Lawson et al., 2022; Quist et al., 2015); (5) and contribution of positive social support experienced in group therapy (Braden et al., 2022; Hesselmark et al., 2014). A sixth mechanism, (6) positive therapeutic alliance, is indicated as a putative mechanism based upon the qualitative information provided (Dandil et al., 2020; Helverschou et al., 2019) but not explicitly discussed within any study.

Approaches to improve emotion regulation varied and included mindfulness and acceptance techniques, increasing interoceptive awareness, psychoeducation, and increasing psychological flexibility. Various outcome measures were used to measure emotion regulation skills, predominantly the *Difficulties in Emotion Regulation Scale* and the *Coping Inventory for Stressful Situations*.

Statistical testing of potential mechanisms was limited. Gaigg et al. (2020) investigated whether intolerance of uncertainty and emotional acceptance were independent predictors of four baseline anxiety measures (*GAD-7, STAI-T, BAI and LSAS*). They suggested that alexithymia may be an underlying risk factor for increased anxiety for autistic people that is expressed through intolerance of uncertainty and emotional acceptance. Analysis in two related studies (Kiep et al., 2015; Spek et al., 2013) suggested that a decline in rumination tendencies mediated the effect of the mindfulness-based protocol on the reduction of anxiety and depression symptoms.

## Objective 4: Adaptations Within Protocols for Autistic Participants (AO Studies)

### Interventions (AO Studies)

Descriptions of intervention protocols varied. These have been synthesized into three groups (Table 5): (1) Amended versions of established protocols, (2) novel psychological interventions (psychological approaches thought to be used for the first time in research with autistic participants), and (3) novel designs (sessions of organized activities or a high dependence on technology to deliver the intervention).

**Table 5.** Studies grouped by intervention protocol, 42 studies with adult participants.

Description	Intervention	Studies
Amended versions of established protocols	CBT based	Flygare et al., 2020; Helverschou et al., 2019; Hesselmark et al., 2014; Ishii et al., 2022; Kuroda et al., 2022; Langdon et al., 2016; Nakagawa et al., 2019; Ordaz et al., 2018; Quist et al., 2015; Sizoo & Kuiper, 2017; Spain et al., 2017; Walhout et al., 2022
	CBT-based guided self-help	Russell et al., 2019
	MBSR based	Beck et al.,2020; Braden et al.,2022; Conner & White 2018; Kiep et al.,2015; Sizoo & Kuiper 2017; Spek et al.,2013
	ACT based	Lawson et al.,2022; Pahnke et al.,2019; Pahnke et al.,2022; Watanabe 2021
	CRT	Dandil et al.,2020; Okuda et al.,2017; Tchanturia et al.,2016
	DBT	Bemmouna et al.,2022; Ritschel et al.,2021
	CBT and DBT-based	Hartmann et al.,2019
	Psychoeducation	Hidalgo et al.,2022; Oswald et al.,2018
	EMDR	Lobregt-van Buuren et al.,2019
Novel psychological interventions	MBSR Psychotherapy + pharmacology	Danforth et al.,2018
	Techniques to notice thoughts	Maisel et al.,2019; Oshima et al.,2021
	CBT-based + brain monitoring	Tsuchiyagaito et al.,2017
	MBSR + neural brain monitoring	Pagni et al.,2020
	CBT-based + virtual reality	Maskey et al.,2019
	Heartbeat detection skills	Brezis et al.,2021; Quadt et al.,2021
Novel designs	Mobile apps encouraging activity	Lee et al.,2022
	Online CBT tools	Gaigg et al.,2020
	Online MBSR tools	Gaigg et al.,2020
	Real-time stress management	Hare et al.,2016
	Emotional prosody identification	Quadt et al.,2021
	Recreation	Hesselmark et al.,2014
	Relaxation	Pagni et al.,2020
	Education & Support	Braden et al.,2022

ACT = acceptance and commitment therapy; CBT = cognitive behaviour therapy; CRT = cognitive remediation therapy; DBT = dialectical behavior therapy; EMDR = eye movement desensitization and reprocessing; MBSR = mindfulness-based stress reduction.

As multiple intervention methods were used within a single study, the number of studies using each method is stated.

*Amended Versions of Established Protocols:* Nine amended versions of established protocols were represented. CBT based protocols were the most used (*n* = 12). The remaining protocols were based on: Mindfulness (MBSR) (*n* = 6); acceptance and commitment therapy (ACT) (*n* = 4); cognitive remediation therapy (CRT) (*n* = 3); dialectical behavior therapy (DBT) (*n* = 2); and psychoeducation (*n* = 2). Protocols used in one study were eye movement desensitization and reprocessing (EMDR); combined CBT and DBT; and guided self-help psychotherapy based on CBT. The use of group and individual therapy formats was featured equally within the studies.

*Novel Psychological Interventions:* Those identified were: Heartbeat detection skills to increase interoceptive awareness (*n* = 2); techniques to notice thoughts (*n* = 2); psychotherapy in conjunction with brain monitoring (*n* = 2); pharmacology (methylenedioxymethamphetamine—MDMA) alongside psychotherapy (*n* = 1); and psychotherapy in conjunction with virtual reality (*n* = 1). There was wide variation in these protocols, ranging from a single, 5-min intervention to teach a cognitive defusion strategy (Maisel et al., 2019) to two 8-h psychotherapy sessions with MDMA, over a 2-month period aimed to reduce symptoms of social anxiety (Danforth et al., 2018).

*Novel Designs:* Novel designs were varied. Hare et al. (2016) used mobile handsets to record questionnaire answers throughout the day. Three studies implemented education, recreation, or relaxation protocols as active comparison conditions (Braden et al., 2022; Hesselmark et al., 2014; Pagni et al., 2020).

To summarize, the most common approach to psychological intervention in the 42 Adult Only studies reviewed was to reuse existing intervention protocols developed for nonautistic people and make amendments thought to be relevant for autistic people. Results for the 27 AC studies are not reported in detail but reflected the same pattern; the majority (*n* = 19) of protocols were based on CBT, MBSR or ACT.

### Adaptations (AO Studies)

**Figure 3.**
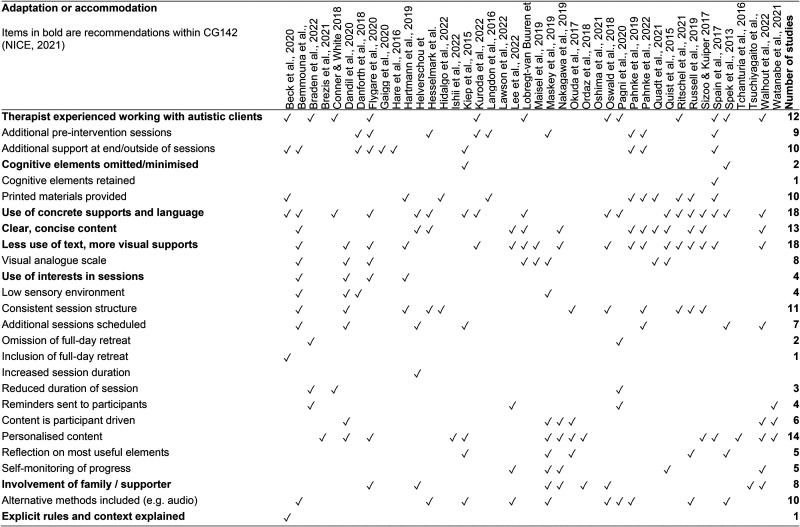
Adaptations for autistic participants documented within 42 studies with adult participants (AO).

Studies tended to provide examples of the adaptations and accommodations made within interventions for autistic participants, rather than exhaustive lists. Therefore, the adaptations (summarized in Figure 3) likely under-represent the full extent of therapy adaptations used within the studies. The most common adaptations included: Use of concrete supports and language (*n* = 18); less use of text and more inclusion of visual supports (*n* = 18); clear and concise content (*n* = 13); and therapists being experienced in working with autistic clients (*n* = 12). Two studies minimized cognitive elements of therapeutic interventions within their protocols, for example, examining one's thoughts was omitted (Kiep et al., 2015; Spek et al., 2013).

The studies represented a range of views about appropriate accommodations for autistic people. For example, two studies omitted a full-day mindfulness retreat usually included in the protocol with nonautistic participants (Braden et al., 2022; Pagni et al., 2020), whereas another study included this, making minimal changes to the protocol (Beck et al., 2020). There were also differences in whether session durations were shortened to avoid overloading participants (Conner & White, 2018) or made longer to support slower processing of concepts (Helverschou et al., 2019). Commonly adopted amendments to CBT protocols (Cooper et al., 2018) were offered in at least a quarter of the studies. Examples included retaining a consistent structure to sessions or offering participants additional support, such as emailing participants as a prompt to undertake self-directed study. Whether adaptations contributed to outcomes was discussed in four studies. The use of visual tools was considered by study authors to be particularly helpful in clarifying abstract concepts and increasing engagement (Dandil et al., 2020; Hartmann et al., 2019; Oswald et al., 2018). Beck et al. (2020) identified two factors they believed were critical: First, interventions being delivered by a teacher experienced in working with autistic people; and second, an additional 30 min of individual support provided to address participant concerns, for example, how to use the audio recordings provided. The variable and sometimes conflicting approaches to adaptations, in the absence of explicit rationale or evaluation, restrict understanding of their usefulness. A key recommendation arising from this review is that adaptations made to protocols are theoretically substantiated and evaluated for their perceived contribution (Spain & Happé, 2020). The analysis identified minimal reporting of the contribution of adaptations to psychological interventions for autistic adults and potential mechanisms of change to wellbeing. The interpretive analysis was therefore limited in its ability to add further insights into how psychological interventions contribute to improved wellbeing for autistic adults.

In summary, 46 of the 69 wellbeing intervention studies included CBT, MBSR, and ACT protocols, usually with amendments considered to suit autistic adults. Studies were predominantly QE pre/post designs with small numbers of participants. There were common RoBs in that participant characteristics were not fully or transparently described. No studies had all outcome measures validated for use with autistic adults. This shows the early stage of research development into wellbeing with autistic adults.

## Discussion

This review maps the current state of evidence for wellbeing interventions for autistic adults. Two key findings are discussed. First, there is a paucity of robust evaluations of psychological wellbeing interventions for autistic adults. Second, the findings identify the high reliance on outcome measures that are not standardized with autistic people.

### Lack of Empirical Research

A key finding of this review was that there is a paucity of robust evaluations of psychological wellbeing interventions for autistic adults. Of the 69 included studies, 42 were pilot or feasibility studies. Sizoo and Kuiper's (2017) comparison of CBT and MBSR-based protocols for symptoms of anxiety and depression was included in the review. This study is often cited as seminal evidence demonstrating the use of psychological therapies with autistic adults (White et al., 2018) despite its relatively small sample size (*n* = 59). As noted in Weston et al.'s (2016) systematic review of CBT interventions with autistic adults and children, the distinction between pilot, feasibility, and empirical studies was blurred, highlighting that valuable exploratory research has not yet advanced to larger-scale evaluations upon which recommendations for psychological intervention can be based. There remains a limited choice of evidence-based psychological interventions for autistic people and practitioners (NICE, 2021). The high number of pilot and exploratory studies may reflect difficulties for researchers in securing funding for projects aimed at improving autistic adults’ wellbeing and related services across the lifespan (Autistica, 2016; IACC, 2018). Of concern is the minimal research funding allocated to projects seeking to understand the needs of autistic adults or improve the quality of autism services (2% and 6% respectively, according to analysis of funding in the United States in 2018 (IACC, 2018)). This contrasts with the majority (63%) of autism research funding allocated to projects investigating the biological mechanisms or “risk factors” underlying autism. As noted in other systematic reviews (Linden et al., 2023; Weston et al., 2016), there were also methodological concerns beyond the lack of evaluative controlled studies, including low study quality, specific risks of bias, and small sample sizes. Collectively, these identify priorities for researchers to address.

Limitations were identified in the representation of autistic people. Younger autistic males were most likely to be included as participants, though it was unclear whether this was due to sampling and selection strategies or a higher number of males with an autism diagnosis (Loomes et al., 2017). Few studies represented gender as a nonbinary construct, yet recent discussions have identified that representation of transgender and gender-diverse identities is important for inclusive practices (Strang et al., 2020; Warrier et al., 2020). Researchers demonstrated their attempts to reconcile the challenge of representing autistic heterogeneity by reporting participants’ clinical characteristics as a rating of autistic “traits”. However, in doing so, these practices reproduce conventions that have been criticized for conceptualizing autism as a continuum and potentially over-representing certain groups of people (den Houting, 2018; D'Mello et al., 2022; Kapp, 2018). We recommend that researchers give fuller descriptions of their samples, including co-occurring neurodivergence and/or mental health need, and medication status (Hobson & Petty, 2021; Lai et al., 2019), rather than relying on static psychometric snapshots of autism (Ashwood et al., 2016). This would also help to develop a better understanding of what the core mechanisms of effective interventions are and how they vary according to individual characteristics or contexts (Elliott et al., 2021; James et al., 2020). For example, analysis of how co-occurring conditions affect response to interventions is warranted (Lai et al., 2019). Challenges remain in capturing the holistic and varied characteristics and phenomenological experiences of autistic individuals in relation to wellbeing outcomes (D'Mello et al., 2022; Heasman & Parfitt, 2023).

Over a third of studies with adult participants also included child or adolescent participants, perhaps highlighting that research is still largely focused on children and younger autistic people (IACC, 2018). Recording changes in behavior or social functioning (e.g., parent-reported decreases in “wandering aimlessly between activities” (Kemeny et al., 2022, p. 2454), “focussing less on the details” or “better eye contact” (de Bruin et al., 2015, p. 911)) were considered an integral part of wellbeing improvement for autistic children in some studies. Children and adolescents vary in how an autism diagnosis is integrated into their developing identity, yet authors did not discuss whether interventions to alter behavior could contribute to conflicting and often negative messages of being different that autistic children navigate (Mesa & Hamilton, 2021). However, it is important to note that this review selected studies with child and adolescent participants based only on their inclusion of adult participants. The studies reviewed here are therefore not representative of wider wellbeing research with autistic children.

This review has built a picture of an emerging field, indicating that clinical research into wellbeing for autistic adults via psychological interventions appears not to have been a priority area. Theoretical conceptualizations of autistic wellbeing frequently lacked personal perspectives of autistic people (Chapman, 2020b; Pearson & Rose, 2021; Rodgers et al., 2020). Interventions were often protocol-driven with minimal opportunities for personal accounts of meaningful outcomes to be explored; there was contradictory use of adaptations without evaluating the contexts in which these might be more effective for some participants; and only a few studies discussed potential mechanisms of change. However, despite the pressing imperative to undertake methodologically robust studies, we caution that a strong underpinning rationale to direct future research is needed first, which must be based on a fit-for-purpose conceptualization of what wellbeing looks like for autistic adults and how it can be influenced. Areas for consideration are discussed next.

### Changing Conceptualizations of Autism

The primary aim of this review was to identify which aspects of psychological interventions have been designed for autistic adults, and what rationale exists for their contribution to improved wellbeing. Specifically, we aimed to understand whether the studies identified the extent to which measures of wellbeing had been designed or confirmed to be suitable for use with autistic people. The findings identify the high reliance on outcome measures that are not standardized with autistic people. Only 14% of measures were documented as having been validated with autistic people. Psychometric outcome measures normed with the general population may be unsuitable for use with autistic people for several reasons (Stewart et al., 2006). First, there are unresolved boundaries between behaviors that are considered to be autistic or reflective of ill-being. For example, social withdrawal might be assumed to be a characteristic of anxiety for a nonautistic person, but conversely may be a positive wellbeing strategy for an autistic person (Uljarević et al., 2018). Second, autistic people may experience mood disorders differently from nonautistic people, showing different forms of emotional distress (Petty et al., 2023b). For example, fatigue caused by sensory and social demands is distinct from anxiety or depression, but equally influential for wellbeing. Third, answering subjective questions about mood is complicated by co-occurrences such as alexithymia (Bird & Cook, 2013). Finally, lists of static behaviors without underpinning explanations over-simplify and constrain personal understanding of items on a psychometric measure (Petty et al., 2023b). Some participants in the studies described questions on psychometric measures as “vague,” “similar,” and “confusing” (Russell, Gaunt et al., 2019, pp. 52–54). An unchallenged assumption across many studies was that symptoms of co-occurring ill-being would be experienced similarly by autistic and nonautistic people. The development of valid scales reflecting autistic perspectives needs to be an ongoing research focus, as exemplified by Bearss et al.'s (2015) development of a scale of anxiety symptoms with autistic youths. In the meantime, we caution against reliance on available outcome measures alone to capture meaningful change within autistic wellbeing intervention research (Brugha et al., 2015).

As well as identifying commonly used outcome measures, this review summarized the available psychological interventions to promote wellbeing. A minority of studies (*n* = 7) followed a medical model conceptualization of autistic characteristics (i.e., had outcomes to reduce core autistic presentations or change behaviors) equating improved well-being to changing autistic “traits,” compared to the majority that focused on various distinct aspects of well-being and mental health outcomes. All but one of these studies had adult and child participants. Social skills training with autistic individuals featured in some studies. This approach to intervention has been criticized for ignoring the two-way and contextual aspects of social interaction, instead placing the onus of responsibility on autistic individuals to alleviate communication difficulties, which can encourage camouflaging behaviors (for a commentary see Bottema-Beutel et al., 2018). Camouflaging behaviors are complex phenomena that can reflect adaptive and resource-intensive responses to contexts (Pearson & Rose, 2021; Petrolini et al., 2023). Camouflaging has been associated with reduced wellbeing for autistic people, for example, through increased exhaustion (Hull et al., 2021; Raymaker et al., 2020). However, a recent review identified that there is wide support from autistic people and stakeholder groups for teaching of *adaptive* skills (Dwyer et al., 2024). We recommend increased critical discussion of the theoretical basis for including social skills training in interventions, including how these relate to personally meaningful adaptive skills and contribute to long-term improvements in wellbeing.

Most studies reviewed were based on existing therapy protocols, with modifications that were thought to accommodate autistic participants’ differences. The most common adaptations aligned with NICE's Clinical Guidance (CG142) and those represented in previous research into clinical practice (Ainsworth et al., 2020; Cooper et al., 2018), such as using concrete supports and language, less use of text, and more inclusion of visual supports. However, differing views about adaptations were discernible, such as whether cognitive demands of protocols should be retained (Spain et al., 2017) or reduced (Kiep et al., 2015; Spek et al., 2013). This highlights the current limited understanding of how adaptations within therapy protocols are delivered and how they might affect change (NICE, 2021).

A recent evaluation of treatment outcomes based on UK healthcare records identified that autistic adults had lower reliable improvements in symptoms of depression or generalized anxiety disorder than the nonautistic comparison group (El Baou et al., 2023). The authors suggested that factors that lead to improved outcomes may not have the same effects for autistic adults as nonautistic people. The exploratory nature of the studies in this review means that we are unable to offer routes to target in wellbeing interventions for autistic adults. Of the six candidate mechanisms identified, emotion regulation was most prominent. Emotion regulation refers to a person's attempts to identify, monitor, and modulate their emotional experience (Gross & Thompson, 2007). Emotion dysregulation has been identified as a risk factor for mental health conditions in the general population, and is considered an important transdiagnostic factor relevant to autistic adults (Cai et al., 2018). Moreover, alexithymia has been associated with emotion dysregulation for nonautistic people (Preece et al., 2023). Studies in this review demonstrated the acceptability of acceptance and cognitive approaches to improve emotion regulation skills, operationalized with two psychometric scales standardized in the general population. However, consensus around outcome measures that reflect framing functions from a neurodiversity perspective could support a more precise approach to understanding potential mechanisms of change (Lukito et al., 2025).

### Implications and Future Research

From the reviewed literature, a range of intervention protocols was considered feasible. However, psychological interventions for autistic people are reliant on outcome measures with unknown suitability to evaluate meaningful change for autistic adults, and a minority of studies equated improved well-being to changing autistic characteristics, demonstrating a degree of conceptual ambiguity of how autism and mental ill health co-occurrences are separable (Chapman & Botha, 2023; Petty et al., 2023a).

The conceptual underpinning of what positive wellbeing would look like for autistic people specifically is missing, further discussion to agree community-based prioritization of the relevance of mental health outcomes is required. Protocols based on CBT, ACT, or MBSR were most common, in both individual and group formats. It remains unknown whether and how amendments to protocols contribute towards outcomes. Despite these limited conclusions, the syntheses reported here provide useful resources for practitioners and care providers. Importantly, this review identifies 35 outcome measures and sub-scales that are validated for use with autistic people (Table 4). Further dialog with autistic stakeholder groups is required to ensure that the outcomes selected for intervention targets are of shared relevance (Pukki et al., 2022).

The review demonstrates the early stage of clinical research into improving wellbeing for autistic adults. We recommend that whilst robust evidence is being sought, practitioners align with person-centered and social justice-informed ways of working with autistic clients (Chapman & Botha, 2023; Petty et al., 2023a) and consider the therapeutic use of phenomenological methods (Green & Shaughnessy, 2023; Pantazakos & Vanaken, 2023). We join many other researchers calling for participatory approaches that foreground autistic production of knowledge (Fletcher-Watson et al., 2019). An example included in the review was Strang et al.'s (2021) Community-Based Participatory Design that iteratively developed a curriculum to identify stakeholders’ support needs with transgender and gender-diverse neurodivergent and autistic adolescents.

There is a pressing need for research focused on building a much better understanding of how wellbeing and ill-being are experienced by autistic people. Emerging conceptual and qualitative explorations suggest potential areas for further research as follows: (1) Development of conceptual frameworks and assessment tools for emotional distress and flourishing that are relevant for autistic people, incorporating, for example, fatigue and exhaustion (Petty et al., 2023b), or autistic burnout (Raymaker et al., 2020); (2) research to understand how environmental and social demands that are not a component of wellbeing can have impacts; for example, the effect of being mis-perceived on self-esteem (Dwyer, 2022; Pukki et al., 2022; Turnock et al., 2022); (3) the development of interventions that support existential growth, for example, awareness of own needs; navigating systems and institutions (Dwyer, 2022; Pukki et al., 2022; Sonuga-Barke, 2023); and (4) evaluating interventions focused on positive contributors to wellbeing, such as building connectedness, positive autistic identity, and understanding the therapeutic value of self-help activities (Botha & Frost, 2020; Cooper et al., 2017; Pukki et al., 2022). These areas reflect contemporary theorizing utilizing a “neurodiversity paradigm translational model” (Sonuga-Barke, 2023, p. 1,406), where the imperative is to design interventions that produce neurodivergent affirmative experiences of personally important strengths (van Os et al., 2019). Individuals’ personal narratives and perspectives of the meaning of wellbeing were notably absent from many studies featured in this review.

### Strengths and Limitations

The review provides the first overview of the current state of wellbeing intervention research with autistic adults that is inclusive of all study designs. Bias was minimized through several processes: The PRISMA protocol, extensive electronic and manual searching, and the use of multiple reviewers. Quality assessment of studies was undertaken via proven tools. The findings draw from studies conducted in multiple geographical locations. Transparency was provided between the data and findings via the synthesis methods used, and the review is therefore replicable.

There are limitations, however. It is difficult to rule out the possibility that there were additional relevant studies not identified through the review process, given the lack of standardized terminology in study titles and abstracts. Only studies published in English were included, which may give a biased view of the current literature. We did not contact study authors to provide further information that was missing from the published study summaries. An unexpected finding was that many studies (39%) included a mix of adult and child participants; it was necessary to separate these from adult-only studies for the syntheses to produce an accurate picture of research with autistic adults. Finally, the review is limited by minimal reporting within the studies of whether salient aspects of interventions (i.e., adaptations to therapy and mechanisms of change) contributed to outcomes.

## Conclusion

This is the first review to indicate the extent and variety of the clinical wellbeing literature for autistic adults. Valuable exploratory research into improving the wellbeing of autistic adults is still at an early stage of development, directed toward demonstrating the feasibility of interventions. Current intervention studies are still reliant on outcome measures with unknown suitability to evaluate meaningful change for autistic adults. Interventions predominantly make use of existing cognitive behavior or mindfulness-based protocols without consensus in best-practice adaptations. A list of outcomes measures validated for use with autistic people has been shared. This review provides a much-needed summary of the academic research literature and provides an essential point of reference for clinical researchers and practising clinicians.

## Supplemental Material

sj-docx-1-dli-10.1177_23969415261436238 - Supplemental material for Fit-for-purpose Psychological Interventions to Support the Well-Being of Autistic Adults: A Systematic Review Supplemental material, sj-docx-1-dli-10.1177_23969415261436238 for Fit-for-purpose Psychological Interventions to Support the Well-Being of Autistic Adults: A Systematic Review by Michele Deakin, Lorna G. Hamilton, Brett Heasman and Stephanie Petty in Autism & Developmental Language Impairments

sj-docx-2-dli-10.1177_23969415261436238 - Supplemental material for Fit-for-purpose Psychological Interventions to Support the Well-Being of Autistic Adults: A Systematic Review Supplemental material, sj-docx-2-dli-10.1177_23969415261436238 for Fit-for-purpose Psychological Interventions to Support the Well-Being of Autistic Adults: A Systematic Review by Michele Deakin, Lorna G. Hamilton, Brett Heasman and Stephanie Petty in Autism & Developmental Language Impairments

sj-docx-3-dli-10.1177_23969415261436238 - Supplemental material for Fit-for-purpose Psychological Interventions to Support the Well-Being of Autistic Adults: A Systematic Review Supplemental material, sj-docx-3-dli-10.1177_23969415261436238 for Fit-for-purpose Psychological Interventions to Support the Well-Being of Autistic Adults: A Systematic Review by Michele Deakin, Lorna G. Hamilton, Brett Heasman and Stephanie Petty in Autism & Developmental Language Impairments

sj-docx-4-dli-10.1177_23969415261436238 - Supplemental material for Fit-for-purpose Psychological Interventions to Support the Well-Being of Autistic Adults: A Systematic Review Supplemental material, sj-docx-4-dli-10.1177_23969415261436238 for Fit-for-purpose Psychological Interventions to Support the Well-Being of Autistic Adults: A Systematic Review by Michele Deakin, Lorna G. Hamilton, Brett Heasman and Stephanie Petty in Autism & Developmental Language Impairments

sj-docx-5-dli-10.1177_23969415261436238 - Supplemental material for Fit-for-purpose Psychological Interventions to Support the Well-Being of Autistic Adults: A Systematic Review Supplemental material, sj-docx-5-dli-10.1177_23969415261436238 for Fit-for-purpose Psychological Interventions to Support the Well-Being of Autistic Adults: A Systematic Review by Michele Deakin, Lorna G. Hamilton, Brett Heasman and Stephanie Petty in Autism & Developmental Language Impairments
