# Supplementary material for: Fit-for-purpose Psychological Interventions to Support the Well-Being of Autistic Adults: A Systematic Review
Source: Autism Dev Lang Impair. 2026 May 15;11:23969415261436238. doi: 10.1177/23969415261436238 (PMC13180214; doi:10.1177/23969415261436238)
Supplement: sj-docx-1-dli-10.1177_23969415261436238 - Supplemental material for Fit-for-purpose Psychological Interventions to Support the Well-Being of Autistic Adults: A Systematic Review [file sj-docx-1-dli-10.1177_23969415261436238.docx]

Supplemental Information 1

Extract Boolean search strings used in all database searches

Data Extract 1 – 18.01.2023

| S8 | S1 AND ( ((S2 OR S3) AND (S4 OR S5)) ) | Limiters - Published Date: 20120101-20231231  Expanders - Apply equivalent subjects  Search modes - Boolean/Phrase | Interface - EBSCOhost Research Databases  Search Screen - Advanced Search  Database - APA PsycInfo;APA PsycArticles;CINAHL with Full Text;MEDLINE |
| --- | --- | --- | --- |
| S7 | S1 AND ( ((S2 OR S3) AND (S4 OR S5)) ) | Expanders - Apply equivalent subjects  Search modes - Boolean/Phrase | Interface - EBSCOhost Research Databases  Search Screen - Advanced Search  Database - APA PsycInfo;APA PsycArticles;CINAHL with Full Text;MEDLINE |
| S6 | S1 AND ( ((S2 OR S3) AND (S4 OR S5)) ) | Expanders - Apply equivalent subjects  Search modes - Boolean/Phrase | Interface - EBSCOhost Research Databases  Search Screen - Advanced Search  Database - APA PsycInfo;APA PsycArticles;CINAHL with Full Text;MEDLINE |
| S5 | S1 AND AB ( depress* OR "low mood" OR "obsessive comp*" OR “obsessive-comp*” OR OCD OR anxiety OR anxious OR stress OR panic OR loneliness OR isolation OR identity OR sleep OR anger OR phobi* OR hypochon* OR anorexia OR eating* OR food OR alcohol OR substance OR "schizo* dis*" OR agoraphob* OR dysmorphia OR PTSD OR “traum*” OR “self harm” OR “self-harm” OR suicid* OR “emotion regulation” OR Quality of life OR Quality-of-life OR QOL OR wellbeing or well-being or “well being” OR “Independent living” OR independent-living OR burnout OR “self worth” OR self-worth ) | Expanders - Apply equivalent subjects  Search modes - Boolean/Phrase | Interface - EBSCOhost Research Databases  Search Screen - Advanced Search  Database - APA PsycInfo;APA PsycArticles;CINAHL with Full Text;MEDLINE |
| S4 | S1 AND TI ( depress* OR "low mood" OR "obsessive comp*" OR “obsessive-comp*” OR OCD OR anxiety OR anxious OR stress OR panic OR loneliness OR isolation OR identity OR sleep OR anger OR phobi* OR hypochon* OR anorexia OR eating* OR food OR alcohol OR substance OR "schizo* dis*" OR agoraphob* OR dysmorphia OR PTSD OR “traum*” OR “self harm” OR “self-harm” OR suicid* OR “emotion regulation” OR Quality of life OR Quality-of-life OR QOL OR wellbeing or well-being or “well being” OR “Independent living” OR independent-living OR burnout OR “self worth” OR self-worth ) | Expanders - Apply equivalent subjects  Search modes - Boolean/Phrase | Interface - EBSCOhost Research Databases  Search Screen - Advanced Search  Database - APA PsycInfo;APA PsycArticles;CINAHL with Full Text;MEDLINE |
| S3 | S1 AND AB ( “practical support” OR “Peer support” OR peer-support OR "self help" OR self-help OR physical OR activity OR psych* OR relax* OR couple* OR collaborat* OR dialect* OR DBT OR remediat* OR "eye movement" OR EMDR OR mindfuln* or mentali* OR "cognitive beh*” OR “cognitive-beh*” OR CBT OR "behav* activ*" OR "interpersonal p*" OR IPT OR “acceptance commitment*” OR ACT OR counsel* OR “behav* therapy” OR person-cent* OR “person cent*” OR "applied behav* analysis" OR ABA OR "applied-behav*-analysis" ) | Expanders - Apply equivalent subjects  Search modes - Boolean/Phrase | Interface - EBSCOhost Research Databases  Search Screen - Advanced Search  Database - APA PsycInfo;APA PsycArticles;CINAHL with Full Text;MEDLINE |
| S2 | S1 AND TI ( “practical support” OR “Peer support” OR peer-support OR "self help" OR self-help OR physical OR activity OR psych* OR relax* OR couple* OR collaborat* OR dialect* OR DBT OR remediat* OR "eye movement" OR EMDR OR mindfuln* or mentali* OR "cognitive beh*” OR “cognitive-beh*” OR CBT OR "behav* activ*" OR "interpersonal p*" OR IPT OR “acceptance commitment*” OR ACT OR counsel* OR “behav* therapy” OR person-cent* OR “person cent*” OR "applied behav* analysis" OR ABA OR "applied-behav*-analysis" ) | Expanders - Apply equivalent subjects  Search modes - Boolean/Phrase | Interface - EBSCOhost Research Databases  Search Screen - Advanced Search  Database - APA PsycInfo;APA PsycArticles;CINAHL with Full Text;MEDLINE |
| S1 | TI Autis* OR asperger* OR ASC OR ASD OR "pervasive dev*" OR PDD OR "high function*" OR HFA | Expanders - Apply equivalent subjects  Search modes - Boolean/Phrase | Interface - EBSCOhost Research Databases  Search Screen - Advanced Search  Database - APA PsycInfo;APA PsycArticles;CINAHL with Full Text;MEDLINE |
