# Supplementary material for: Fit-for-purpose Psychological Interventions to Support the Well-Being of Autistic Adults: A Systematic Review
Source: Autism Dev Lang Impair. 2026 May 15;11:23969415261436238. doi: 10.1177/23969415261436238 (PMC13180214; doi:10.1177/23969415261436238)
Supplement: sj-docx-2-dli-10.1177_23969415261436238 - Supplemental material for Fit-for-purpose Psychological Interventions to Support the Well-Being of Autistic Adults: A Systematic Review [file sj-docx-2-dli-10.1177_23969415261436238.docx]

Supplemental Information 4

Participant characteristics for 69 included studies

| **Authors** | **Intervention** | ***N=*** | **M** | **F** | **O** | **M(Age)** | **SD** | **Age**  **Range** |
| --- | --- | --- | --- | --- | --- | --- | --- | --- |
| **42 A Studies** |  |  |  |  |  |  |  |  |
| Braden et al., 2022 | MBSR based | 28 | 16 | 12 |  | 30.32 | 11.74 | 18-64 |
|  | Education/support | 27 | 18 | 9 |  | 32.56 | 14.83 | 18-72 |
| Danforth et al., 2018 | Psychotherapy + MDMA | 8 | 6 | 2 |  | 32.80 | 10.40 | NR |
|  | Psychotherapy + placebo | 4 | 4 | 0 |  | 28.30 | 3.80 | NR |
| Gaigg et al., 2020 | MBSR online tools | 14 | 12 | 2 |  | 42.50 | 10.30 | 28–66 |
|  | CBT online tools | 9 | 8 | 1 |  | 40.30 | 12.70 | 26–58 |
|  | Waitlist | 16 | 12 | 4 |  | 45.70 | 13.60 | 23–64 |
| Hesselmark et al., 2014 | CBT based | 34 | 17 | 17 |  | 31.90 | 8.50 | NR |
|  | Recreational activity | 34 | 24 | 10 |  | 31.80 | 9.60 | NR |
| Kuroda et al., 2022 | CBT based | 29 | 19 | 10 |  | 32.70 | 8.10 | NR |
|  | Waitlist | 29 | 21 | 8 |  | 29.60 | 8.00 | NR |
| Langdon et al., 2016 | CBT + TAU | 26 | 12 | 14 |  | 33.10 | 14.60 | 20-64 |
|  | Waitlist | 26 | 15 | 11 |  | 38.70 | 14.30 | 17-65 |
| Lee et al., 2022 | Physical activity, app 1 | 12 | 3 | 9 |  | 27.10 | 7.50 | NR |
|  | Physical activity, app 2 | 12 | 6 | 6 |  | 31.90 | 11.30 | NR |
| Maisel et al., 2019 | Cog defusion /distraction  (autistic) | 42 | 27 | 15 |  | 24.36 | 6.18 | 18-47 |
|  | Reported as neurotypical | 55 | 41 | 14 |  | 21.36 | 2.08 | 17-27 |
| Oswald et al., 2018 | CBT + psychoeducation | 25 | 16 | 9 |  | 24.90 | 6.10 | 18-38 |
|  | Waitlist | 16 | 12 | 4 |  | 25.50 | 7.10 | 18-38 |
| Pagni et al., 2020 | MBSR based+neural mon | 15 | 8 | 7 |  | 32.37 | 13.16 | 18-64 |
|  | Relaxation+neural mon | 13 | 11 | 2 |  | 31.15 | 13.22 | 19.62 |
| Pahnke et al., 2022 | ACT | 20 | 10 | 10 |  | 38.40 | 10.00 | 21-72 |
|  | TAU | 19 | 11 | 8 |  | 39.80 | 14.40 | 21-72 |
| Quadt et al., 2021 | Interoceptive +MBSR | 61 | 33 | 26 | 2 | 29.00 | NR | 18-64 |
|  | Prosody | 60 | 32 | 24 | 4 | 31.00 | NR | 19-59 |
| Russell et al., 2019 | CBT guided self-help | 34 | 24 | 10 |  | 35.30 | 13.60 | NR |
|  | TAU | 35 | 27 | 8 |  | 40.20 | 12.60 | NR |
| Spek et al., 2013 | MBSR based | 20 | 13 | 7 |  | 44.40 | 11.10 | NR |
|  | WL | 21 | 14 | 7 |  | 40.10 | 11.00 | NR |
| Nakagawa et al., 2019 | CBT based (non autistic) | 18 | 4 | 14 |  | 44.78 | 11.10 | NR |
|  | CBT based (autistic) | 12 | 7 | 5 |  | 39.42 | 11.06 | NR |
| Sizoo & Kuiper, 2017 | CBT based | 27 | 19 | 8 |  | 35.10 | 9.22 | NR |
|  | MBSR based | 32 | 19 | 13 |  | 39.40 | 10.81 | NR |
| Tsuchiyagaito et al., 2017 | CBT based (autistic) | 15 | 11 | 4 |  | 29.53 | 7.25 | NR |
|  | CBT based (non autistic) | 22 | 5 | 17 |  | 34.09 | 7.30 | NR |
| Lobregt-van Buuren et al., 2019 | EMDR + TAU | 21 | 13 | 8 |  | 34.48 | 11.73 | NR |
|  | Waitlist + TAU |  |  |  |  |  |  |  |
| Beck et al., 2020 | MBSR based | 12 | 9 | 3 |  | 37.58 | 14.04 | 22-63 |
| Bemmouna et al., 2022 | DBT | 7 | 4 | 3 |  | 27.71 | 13.34 | 19-56 |
| Brezis et al., 2021 | Biofeedback | 14 | 12 | 2 |  | 29.71 | 8.44 | 19-48 |
| Conner & White, 2018 | MBSR based | 9 | 7 | 2 |  | 19.56 | 2.19 | 18-25 |
| Flygare et al., 2020 | CBT based | 19 | 8 | 11 |  | 23.84 | 5.90 | NR |
| Hartmann et al., 2019 | CBT/DBT based | 7 | 4 | 3 |  | 21.00 | 2.60 | 18-24 |
| Helverschou et al., 2019 | CBT based | 4 | 4 | 0 |  | 31.40 | NR | 22- 44 |
| Hidalgo et al., 2022 | Psychoeducation | 71 | 40 | 31 |  | 32.70 | 12.00 | 18-64 |
| Ishii et al., 2022 | CBT based | 10 | 10 | 0 |  | 27.40 | 5.60 | NR |
| Kiep et al., 2015 | MBSR based | 50 | 34 | 16 |  | 40.00! | 12.25 |  |
| Lawson et al., 2022 | ACT | 8 | 6 | 2 |  | NR | NR | 18-70 |
| Okuda et al., 2017 | CRT | 16 | 12 | 4 |  | 29.56 | 11.00 | 18-49 |
| Oshima et al., 2021 | Schema therapy | 10 | 5 | 5 |  | 26.80 | 6.39 | 20-39 |
| Pahnke et al., 2019 | ACT | 10 | 5 | 5 |  | 49.00 | 12.00 | 25-65 |
| Quist et al., 2015 | CBT based psychoed | 14 | 14 | 0 |  | 28.16 | NR | 20-55 |
| Ritschel et al., 2021 | DBT | 16 | 12 | 4 |  | 36.62 | NR | 19-68 |
| Spain et al., 2017 | CBT based | 14 | 14 | 0 |  | 31.00 | 7.90 | 22-48 |
| Walhout et al., 2022 | CBT based | 30 | 26 | 4 |  | 41.10 | 12.84 | 24-64 |
| Dandil et al., 2020 | CRT | 1 | 0 | 1 |  | 21.00 | - | - |
| Hare et al., 2016 | RTSM | 9 | 5 | 4 |  |  |  |  |
| Maskey et al., 2019 | CBT based +virtual reality | 8 | 4 | 4 |  | 29.80 | NR | 18-57 |
| Ordaz et al., 2018 | CBT based | 1 |  | 1 |  | 18.00 |  |  |
| Tchanturia et al., 2016 | CRT based (low ‘traits’) | 21 |  | NR | 21 | 26.20 | 7.70 | NR |
|  | CRT based (high ‘traits’) | 14 | NR | NR | 14 |  |  |  |
| Watanabe 2021 | ACT | 1 | 0 | 1 |  | 42.00 | NR | NA |
| **AO Totals** |  | **1267** | **785** | **441** | **41** |  |  |  |
| AO Mean of Means Age |  |  |  |  |  | 32.66 |  |  |
|  |  |  |  |  |  |  |  |  |
| **27 AC Studies** |  |  |  |  |  |  |  |  |
| Capriola-Hall et al., 2021 | CBT based | 16 | 11 | 5 |  | 19.87 | 1.92 | 16-25 |
|  | TAU | 16 | 13 | 3 |  | 19.63 | 2.25 | 16-25 |
| Murphy et al., 2017 | CBT based | 17 | 10 | 7 |  | 14.94 | 1.63 | 12-18 |
|  | Counselling | 19 | 12 | 7 |  | 15.56 | 1.91 | 12-18 |
| Russell et al., 2013 | CBT for OCD | 23 | 19 | 4 |  | 28.60 | 11.30 | 14-49 |
|  | Anxiety management | 23 | 16 | 7 |  | 25.20 | 13.50 | 14-65 |
| Wolters et al., 2016 | CBT based | 36 | 14 | 22 |  | 12.80 | 2.60 | 8-18 |
|  | WL | 22 | 10 | 12 |  | 13.00 | 2.60 | 8-18 |
| Yang & Chung, 2022 | CBT App | 15 | 13 | 2 |  | 21.47 | 5.33 | 15-35 |
|  | WL | 15 | 14 | 1 |  | 20.47 | 4.89 | 15-35 |
| McGillivray & Evert, 2014 | CBT based | 26 | 19 | 7 |  | 20.27 | 4.39 | 15-25 |
|  | WL | 16 | 13 | 3 |  | 20.50 | 3.40 | 15-25 |
| Murray et al., 2015 | CBT based (autistic) | 22 | 14 | 8 |  | 15.00 | 3.25 | NR |
|  | CBT based (non autistic) | 22 | 12 | 10 |  | 15.00 | 4.25 | NR |
| van Steensel et al., 2014 | CBT based | 24 | 20 | 4 |  | 11.00 | 2.62 | 8-18 |
|  | TAU | 25 | 20 | 5 |  | 10.72 | 2.25 | 8-18 |
| Zaharia et al., 2021 | Education | 14 | 14 | 0 |  | 17.79 | 6.52 | 10-35 |
|  | WL | 16 | 16 | 0 |  | 18.44 | 6.37 | 10-35 |
| Ekman & Hiltunen, 2015 | CBT based (Adults) | 7 | 4 | 3 |  | 29.80 | 4.40 | 23-36 |
|  | CBT based (Teens) | 11 | 7 | 4 |  | 14.90 | 1.50 | 13-17 |
| Kemeny et al., 2022 | MBSR based | 27 | 20 | 7 |  | 16.33 | 2.77 | 12-21 |
|  | Horse riding | 0 |  |  |  |  |  |  |
|  | WL | 0 |  |  |  |  |  |  |
| Pahnke et al., 2014 | ACT | 15 | 9 | 6 |  | 16.20 | 1.40 | 13-21 |
|  | WL | 13 | 12 | 1 |  | 16.80 | 2.50 | 13-21 |
| Backman et al., 2018 | Psychoeducation | 28 | 12 | 16 |  | 20.62 | 2.60 | 16-25 |
| Bemmer et al., 2021 | CBT based | 69 | 39 | 29 | 1 | 22.30 | 4.70 | 16-33 |
| Brandsma et al., 2022 | Peer support | 41 | 23 | 18 |  | 17.80 | 2.32 | NR |
| Carey et al., 2022 | Physical activity | 24 | 24 | 0 |  | 10.79 | 3.87 | 5-18 |
| Connor et al., 2020 | Psychoeducation | 26 | 21 | 5 |  | 20.40 | 1.50 | 17-23 |
| de Bruin et al., 2015 | MBSR based | 23 | 17 | 6 |  | 15.08 | 2.70 | 11-23 |
| Jackson et al., 2022 | Physical activity | 11 | 9 | 1 | 1 | 14.70 | 6.60 | 7-24 |
| Mahler et al., 2022 | Interoception training | 14 | 11 | 3 |  | 13.86 | 3.30 | 9-19 |
| Reaven et al., 2012 | CBT based | 24 | 15 | 9 |  | 15.50 | NR | 13-18 |
| Ridderinkhof et al., 2018 | MBSR based | 45 | 36 | 9 |  | 13.03 | 2.72 | 8-19 |
| Salem-Guirgis et al., 2019 | MBSR based | 23 | 19 | 4 |  | 15.65 | 2.57 | 12-23 |
| Wise et al., 2019 | CBT based | 7 | 4 | 3 |  | 17.14 | 1.68 | 16-20 |
| Ehrenreich-May et al., 2020 | CBT based | 6 | 5 | 1 |  | 16.50 |  | 15-21 |
| Ridderinkhof et al., 2021 | MBSR based | 5 | 4 | 1 |  | 16.60 | NR | 14-18 |
| Strang et al., 2021 | Peer Support + education | 31 | 0 | 0 | 31 | 15.92 | 1.85 | 12-19 |
| **AC Totals** |  | **817** | **551** | **233** | **33** |  |  |  |

!Kiep et al., 2015 males (mean age 42.1; SD 10.5) and females (mean age 37.9; SD 14).

ACT: acceptance and commitment therapy; CBT: cognitive behaviour therapy; CRT: cognitive remediation therapy; DBT: dialectical behaviour therapy; EMDR: eye movement desensitisation and reprocessing; F: female; M: male; M(Age): mean age; MDMA: methylenedioxy methamphetamine; MBSR: mindfulness based stress reduction; OCD: obsessive compulsive disorder; O: other; QE: quasi-experimental; QoL: quality of life; SD: standard deviation; TAU: treatment as usual.
