# Supplementary material for: Fit-for-purpose Psychological Interventions to Support the Well-Being of Autistic Adults: A Systematic Review
Source: Autism Dev Lang Impair. 2026 May 15;11:23969415261436238. doi: 10.1177/23969415261436238 (PMC13180214; doi:10.1177/23969415261436238)
Supplement: sj-docx-3-dli-10.1177_23969415261436238 - Supplemental material for Fit-for-purpose Psychological Interventions to Support the Well-Being of Autistic Adults: A Systematic Review [file sj-docx-3-dli-10.1177_23969415261436238.docx]

Supplemental Information 3

Conceptual map of 42 studies with adult participants (AO)

**
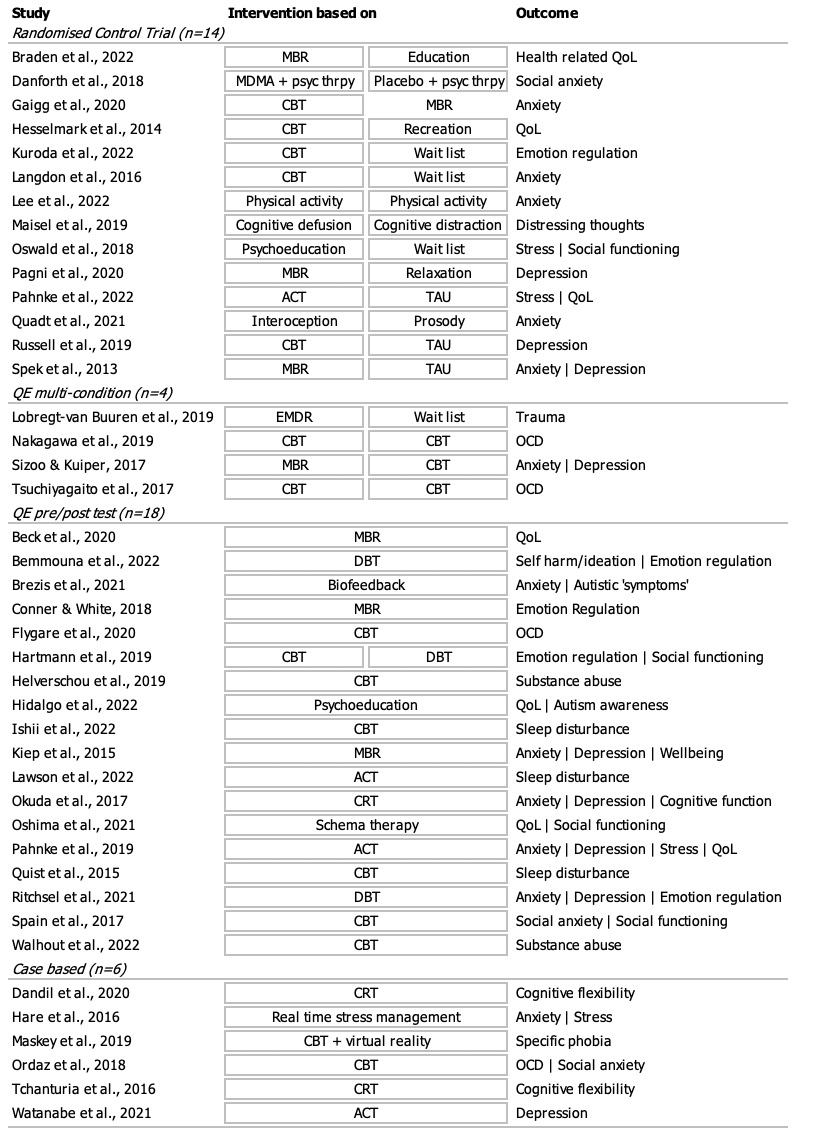
**

ACT: acceptance and commitment therapy; CBT: cognitive behaviour therapy; CRT: cognitive remediation therapy; DBT: dialectical behaviour therapy; EMDR: eye movement desensitisation and reprocessing; MDMA: methylenedioxy methamphetamine; MBR: mindfulness-based stress reduction; OCD: obsessive compulsive disorder; QE: quasi-experimental; QoL: quality of life.

Conceptual map of 27 studies with child and adult participants (AC)

**
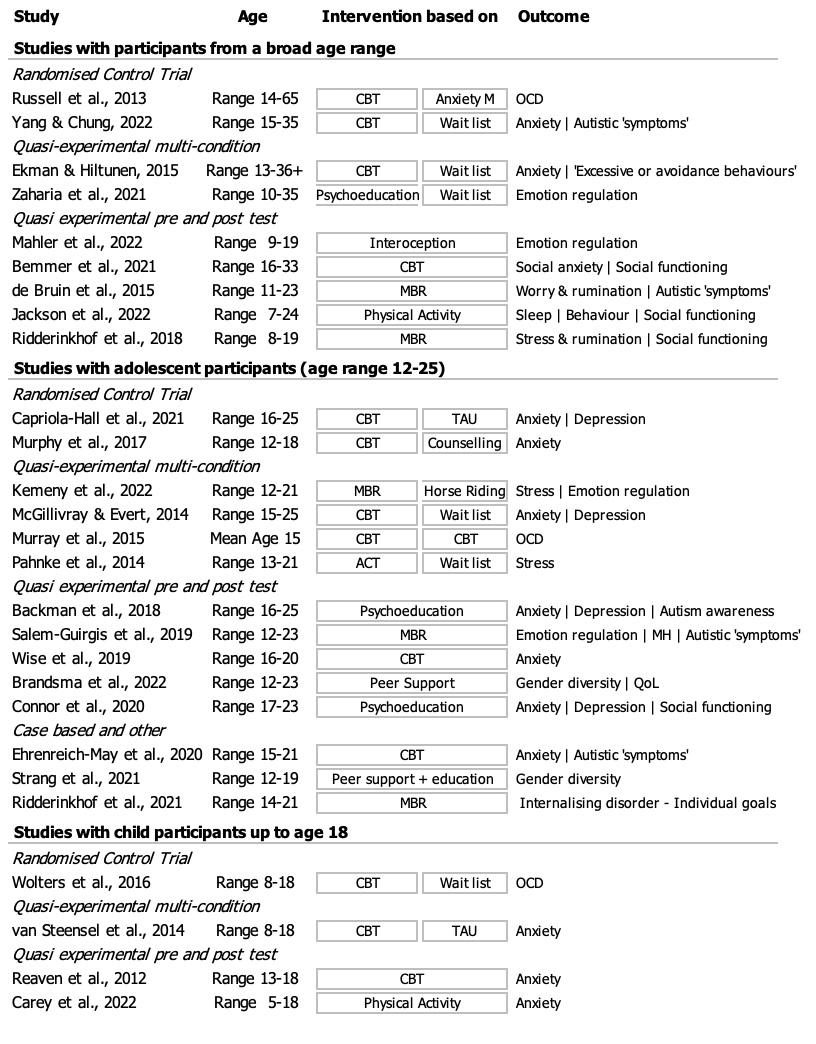
**

ACT: Acceptance and commitment therapy; CBT: Cognitive behaviour therapy; MBR: Mindfulness-based stress reduction; OCD: obsessive compulsive disorder; QoL: Quality of Life; TAU: treatment as usual.
