# Supplementary material for: Fit-for-purpose Psychological Interventions to Support the Well-Being of Autistic Adults: A Systematic Review
Source: Autism Dev Lang Impair. 2026 May 15;11:23969415261436238. doi: 10.1177/23969415261436238 (PMC13180214; doi:10.1177/23969415261436238)
Supplement: sj-docx-4-dli-10.1177_23969415261436238 - Supplemental material for Fit-for-purpose Psychological Interventions to Support the Well-Being of Autistic Adults: A Systematic Review [file sj-docx-4-dli-10.1177_23969415261436238.docx]

Supplemental Information 2

*Detailed risk of bias synthesis based on adapted Wigham et al., (2017) risk of bias tool*

| Study  (order: AO then AC) | Verification of participant’s autism diagnosis | Description  of participants | Description of recruitment | Outcome measure | the general  Reported | Outcome  population  Study RoB | measure validity  autistic people  Reported | RoB | Study RoB |
| --- | --- | --- | --- | --- | --- | --- | --- | --- | --- |
| Beck et al., 2020 | **LR** | **SC** | **LR** | WASI-Wechsler Abbreviated Scale of Intelligence  CSQ-8 Client Satisfaction Questionnaire  PRPS Pittsburgh Rehabilitation and Participation Rating Scale  UOMS Understanding of Material Scale  SWLS Satisfaction with Life, Global QOL satisfaction and well-being  WHOQOL-BREF-ID World Health Organisation (WHO) QoL  WHOQOL-DIS-ID WHO Disability  CAMM Child and Adolescent Mindfulness Measure  HEAL Healing Encounters and Attitudes Lists, Positive Outlook Scale | NR  Yes  Yes  NR  NR  NR  NR  NR  Yes | NA | NR  NR  Used previously  Used previously  Yes  Yes  Yes  Yes  NR | HR  HR  SC  SC  LR  LR  LR  LR  HR | **SC** |
| Bemmouna et al., 2022 | **LR** | **LR** | **LR** | WAIS-IV Wechsler Adult Intelligence Scale, Fourth Edition  CSQ-8 Client Satisfaction Questionnaire for psychotherapeutic services  DERS Difficulties in Emotion Regulation  BHS Beck Hopelessness Scale  GAFS-8 General Alexithymia Factor Score  BDI-II Beck Depression Inventory, Second Edition  WHOQoL-BREF Abbreviated WHO QOL Questionnaire | NR  Yes  Yes  Yes  NR  Yes  Yes | NA | NR  NR  Yes  Unknown  Yes  Yes  Yes | HR  HR  LR  HR  LR  LR  LR | **SC** |
| Braden et al., 2022 | **LR** | **SC** | **SC** | WHOQOL-BREF / DAS / AS WHO QoL Brief / Disability / Autistic Spectrum Version  KBIT-2 Kaufman brief intelligence test (second edition)  WHODAS WHO Disability Assessment Schedule | NR | NA | NR | HR | **HR** |
| Brezis et al., 2021 | **SC** | **SC** | **SC** | WAIS Vocabulary and Matrices subscales  SRS Social Responsiveness Scale Autism Symptoms  GAS Glasgow Anxiety Scale, Anxiety  TAS-20 Toronto Alexithymia Scale  ARC’s self-determination scale  RMET Reading the Mind in the Eyes Test, Cognitive empathy  Response to Challenging Situations – interview | NR | NA | NR | HR | **HR** |
| Conner & White, 2018 | **SC** | **LR** | **HR** | WAIS-II Wechsler Abbreviated Scale of Intelligence, Second Edition  MINI Mini International Neuropsychiatric Interview  DERS Difficulties in Emotion Regulation Scale  OQ Outcome Questionnaire | NR  NR  Yes  Yes | NA | NR | HR | **HR** |
| Dandil et al., 2020 | **LR** | **SC** | **LR** | DFlex The Detail and Flexibility Questionnaire  ROCF The Rey–Osterrieth Complex Figure  The Brixton Spatial Anticipation Test  EDE-Q Eating Disorder Examination  Motivational ruler – visual analogue scales | NR | NA | NR | HR | **HR** |
| Danforth et al., 2018 | **LR** | **LR** | **LR** | LSAS Leibowitz Social Anxiety Scale  SCID-I-RV Structured Clinical Interview for Diagnostic and Statistical  Manual of Mental Disorders- Fourth Edition Axis  C-SSRS Columbia Suicide Severity Rating Scale  BDI-II Beck Depression Inventory  PSS Perceived Stress Scale  IRI Interpersonal Reactivity Index  RSES Rosenberg Self-Esteem Scale  STAI From Y-2 Spielberger State-Trait Inventory  TAS-20 Toronto Alexithymia Scale  TASIT The Awareness of Social Inference Test  ERQ Emotion Regulation Questionnaire | NR | NA | LSAS -Used previously  NR for other measures | SC  HR  HR  HR  HR  HR  HR  HR  HR  HR  HR | **SC** |
| Flygare et al., 2020 | **LR** | **SC** | **LR** | OCI-R Obsessive Compulsive Inventory–Revised  MINI Mini-International Neuropsychiatric Interview  Y-BOCS Symptom Checklist  CGI-S Clinical Global Impression Scale for severity  CGI-I Clinical Global Impression Scale for improvement  GAF Global Assessment of Functioning  AUDIT Alcohol use disorders identification test  DUDIT Drug use disorders identification test  MADRS-S Montgomery Asberg Depression Rating Scale–Self report  EQ-5D EuroQol 5-dimensions | NR  NR  Yes  NR  NR  NR  NR  NR  Yes  Yes | NA | OCI-R – validated  NR for other measures. | LR  HR  HR  HR  HR  HR  HR  HR  HR  HR | **SC** |
| Gaigg et al., 2020 | **LR** | **SC** | **LR** | WAIS-III-UK Wechsler Adult Intelligence Scale or  WAIS-IV Wechsler Adult Intelligence Scale  BAI Beck’s Anxiety Inventory  LSAS Liebowitz Social Anxiety Scale  HADS-D Depression Hospital Anxiety and Depression Scale  CORE-OM Clinical Outcomes in Routine Evaluation – Outcome Measure  GAD-7 The General Anxiety Disorder-7  STAI-T State-Trait Anxiety Inventory  IUS-12 Intolerance of Uncertainty Scale  FFMQ-NR Five Facet Mindfulness Questionnaire ,  non-reactivity to inner experiences sub-scale  BVAQ-ID The Bermond-Vorst Alexithymia Questionnaire ,  sum of the Identify and Describe subscales | NR | NA | NR | HR  HR  HR  HR  HR  HR  HR  HR  HR  HR  HR | **HR** |
| Hare et al., 2016 | **LR** | **SC** | **SC** | WASI Wechsler Abbreviated Scale of Intelligence  BPVS II The British Picture Vocabulary Scale  HADS Hospital Anxiety and Depression Scale  PDA questions (developed for previous AS study) | NR | NA | NR  NR  Used previously  Used previously | HR  HR  SC  SC | **SC** |
| Hartmann et al., 2019 | **LR** | **SC** | **SC** | WASI-II Wechsler Abbreviated Scale of Intelligence II  SRS-2 The Social Responsiveness Scale, Second Edition  ERQ Emotion Regulation Questionnaire  SPAI-23 The Social Phobia and Anxiety Inventory-23  BPAQ The Buss and Perry Aggression Questionnaire | NR | NA | NR | HR  HR  HR  HR  HR | **HR** |
| Helverschou et al., 2019 | **LR** | **SC** | **LR** | WAIS-III or WAIS Wechsler Abbreviated Scale of Intelligence  HSCL-25 Hopkins Symptom Checklist-25  DUDIT-E Drug Use Disorder Identification Test – Extended  AUDIT-E Alcohol Use Disorders Identification Test – Extended  GAF The Global Assessment of Functioning  MI The Motivational Interview  PSQ-18 Patient Satisfaction Questionnaire | Yes (for all) | NA | HSCL used with several populations.  NR for others. | HR  HR  HR  HR  HR  HR  HR | **HR** |
| Hesselmark et al., 2014 | **LR** | **SC** | **LR** | QOLI The QoL Inventory  SoC Sense of Coherence Scale  RSES Rosenberg Self-Esteem Scale  SCL-90 Symptom Checklist 90  AQ Autism Quotient  BDI Beck Depression Inventory  ASRS Adult ADHD Self-Report Scale  CGI-S Clinical Global Impression Scale, Severity  CGI-I Clinical Global Impression Scale, Improvement | NR | NA | NR | HR  HR  HR  HR  HR  HR  HR  HR  HR | **HR** |
| Hidalgo et al., 2022 | **LR** | **SC** | **LR** | TCS – Treatment Credibility Scale  ASD 20 Questions (knowledge quiz about Autism)  QAFM Questions About Family Members  HADS The Hospital Anxiety and Depression Scale , Depression subscale  HADS The Hospital Anxiety and Depression Scale, Anxiety sub scale  SWLS Satisfaction With Life Scale  Acceptance and Action Questionnaire – II  BAS Burden Assessment Scale | NR | NA | NR | HR  HR  HR  HR  HR  HR  HR  HR | **HR** |
| Ishii et al., 2022 | **HR** | **SC** | **LR** | ISI Insomnia severity index  HADS Hospital Anxiety and Depression Scale, Depression subscale  HADS Hospital Anxiety and Depression Scale, Anxiety subscale  ASRS ADHD Self‐Report Scale, Inattentive subscale  ASRS ADHD Self‐Report Scale, Hyperactive/Impulsive subscale | NR | **HR** | NR | HR  HR  HR  HR  HR | **HR** |
| Kiep et al., 2015 | **LR** | **SC** | **LR** | WAIS-III Wechsler Adult Intelligence Scale III  SCL-90-R Symptom Checklist-90-Revised  RRQ Rumination-Reflection Questionnaire, Rumination subscale  GMS Dutch Global Mood scale, Positive affect subscale | NR  Yes  Yes  Yes | NA | NR | HR  HR  HR  HR | **HR** |
| Kuroda et al., 2022 | **SC** | **SC** | **LR** | WAIS-III Wechsler Adult Intelligence Scale, Third Edition, Japanese version  CISS Coping Inventory for Stressful Situations  TAS-20 Toronto Alexithymia Scale Japanese version  MPMR Motion Picture Mind-Reading Task  ASD-Q ASD Knowledge and Attitude Quiz  GAF Global Assessment of Functioning  WHO QoL World Health Organization Quality of Life Scale  LSAS Liebowitz Social Anxiety Scale  STAI State-Trait Anxiety Inventory  SPAI Social Phobia and Anxiety Inventory  CES-D Center for Epidemiologic Studies Depression Scale | NR | NA | NR | HR  HR  HR  HR  HR  HR  HR  HR  HR  HR  HR | **HR** |
| Langdon et al., 2016 | **SC** | **SC** | **LR** | WAIS Wechsler Adult Intelligence Scale  HAM-A Hamilton Rating Scale for Anxiety  Social Phobia Inventory  LSAS Liebowitz Social Anxiety Scale  Social and Emotional Functioning Interview  Social Interaction Anxiety Scale  Fear Questionnaire  HAM-D Hamilton Rating Scale for Depression  EuroQol EQ-5D Generic health-related quality of life | NR | NA | NR | HR  HR  HR  HR  HR  HR  HR  HR  HR | **HR** |
| Lawson et al., 2022 | **LR** | **HR** | **SC** | ISI Insomnia Severity Index  HADS Hospital Anxiety and Depression Scale  PSQI Pittsburgh Sleep Quality Index  CORE-10 short measure of psychological distress  BEAQ Brief Experiential Avoidance Questionnaire  SAAQ Sleep Anticipatory Anxiety Questionnaire  FFS Flinders Fatigue Scale | Yes  Yes  NR  Yes  Yes  Yes  Yes | NA | NR  Yes  Yes  NR  NR  Yes  Yes | HR  LR  LR  HR  HR  LR  LR | **SC** |
| Lee et al., 2022 | **SC** | **SC** | **SC** | AQ-10 Autism Spectrum Quotient, 10 item questionnaire  BAI The Beck Anxiety Inventory  Daily Anxiety Assessment (Specific for this intervention) | Yes  NA | NA | NR | HR  HR  HR | **HR** |
| Lobregt-van Buuren et al., 2019 | **LR** | **LR** | **LR** | IES-R The Impact of Event Scale-Revised  Adapted Anxiety Disorders Interview Schedule-Children (ADIS-C) section  PTSD version for adults with mild to borderline intellectual disabilities  BSI Brief Symptom Inventory, short version of SCL-90-R  SRS-A Social Responsiveness Scale-Adult | NR  Yes  Yes  Yes | NA | NR  Part-validated  NR  Yes | HR  SC  HR  LR | **SC** |
| Maisel et al., 2019 | **LR** | **SC** | **LR** | AQ The Autism Spectrum Quotient  DASS-21 The Depression Anxiety and Stress Scale-21  CFQ The Cognitive Fusion Questionnaire  BAFT The Believability of Anxious Feelings and Thoughts Questionnaire  Subjective Measures: To assess subjective thought believability and  discomfort, participants were shown a Visual Analogue Scale | Yes  Yes  Yes  Yes  NA | **LR** | Yes  NR  Part-validated  NR  NR | LR  HR  SC  HR  HR | **SC** |
| Maskey et al., 2019 | **LR** | **SC** | **LR** | SRS-2 Social Responsiveness Scale-second edition  BAI Beck Anxiety Inventory  GAD-7 Generalized Anxiety Disorder 7  PHQ-9 Patient Health Questionnaire-9  WHOQOL-BREF WHO Quality of Life  Self-report ratings of participants’ confidence in managing the target  anxiety situation were taken using a 6-point visual analogue scale | NR | NA | Yes  NR | LR  HR  HR  HR  HR  HR | **SC** |
| Nakagawa et al., 2019 | **LR** | **SC** | **SC** | SCID-IV Structured Clinical Interview for the Diagnostic and Statistical  Manual of Mental Disorders, version IV  Wechsler Intelligence Scale  Y-BOCS Yale-Brown Obsessive Compulsive Scale  BDI-II Beck Depression Inventory-Second Edition | NR  NR  Yes  Yes | **LR** | NR | HR  HR  HR  HR | **HR** |
| Okuda et al., 2017 | **LR** | **SC** | **SC** | WAIS-III Wechsler Adult Intelligence Scale  The Brixton Test  TMT Trail-making Test,  Stroop Test  WCST (Keio version)  ROCF Rey–Osterrieth Complex Figure Test  GAD-7 The General Anxiety Disorder-7  PHQ-9 Patient Health Questionnaire  HADS Hospital Anxiety and Depression Scale 7  RSES Rosenberg Self-esteem Scale  CFS Cognitive Flexibility Scale  EQ-5D EuroQol 5 dimensions | NR | NA | NR | HR  HR  HR  HR  HR  HR  HR  HR  HR  HR  HR  HR | **HR** |
| Ordaz et al., 2018 | **LR** | **LR** | **SC** | ADIS-5 Anxiety and Related Disorders Interview Schedule  Y-BOCS Yale–Brown Obsessive Compulsive Scale Symptom Checklist  CGI-S Clinical Global Impression of Severity  WASI-II Wechsler Abbreviated Scale of Intelligence–Second Edition  BAI Beck Anxiety Inventory  CGI-I Clinical Global Impression of Improvement  CAIS-P Child Anxiety Impact Scale–Parent  FAS-A Family Accommodation Scale for Anxiety | NR | NA | NR | HR  HR  HR  HR  HR  HR  HR  HR | **HR** |
| Oshima et al., 2021 | **HR** | **SC** | **SC** | WAIS-III Wechsler Adult Intelligence Scale III  MINI-International Neuropsychiatric Interview  GAF Global Assessment Functioning  WHO QOL-BREF World Health Organization quality of life assessment brief  SMI Schema Mode Inventory  YSQ-SF Young Schema Questionnaire-Short Form 3  BDI- II Beck Depression Inventory II  STAI State-Trait Anxiety Inventory  LSAS Liebowitz Social Anxiety Scale | NR | NA | NR | HR  HR  HR  HR  HR  HR  HR  HR  HR | **HR** |
| Oswald et al., 2018 | **LR** | **SC** | **LR** | WASI-II Wechsler Abbreviated Scale of Intelligence—Second Edition  ABAS-3 Adaptive Behavior Assessment System, Adult Form, third Edition  Seven Component Self-Determination Skills Survey.  CSES Coping Self-Efficacy Scale  ASR ASEBA Adult Self-Report | NR  Yes  NR  Yes  Yes | NA | Yes  NR  Validated with ID  NR  NR | LR  HR  SC  HR  HR | **SC** |
| Pagni et al., 2020 | **LR** | **SC** | **HR** | KBIT-2 Kaufman Brief Intelligence Test-2  BDI-II Beck Depression Inventory-2  STAI State-Trait Anxiety Inventory | NR | NA | NR | HR  HR  HR | **HR** |
| Pahnke et al., 2019 | **LR** | **SC** | **LR** | WAIS-R or the WAIS-III  CPT-II Conner's Continuous Performance Test and/or  D-KEFS Delis-Kaplan Executive Function System  TSC ASD adapted version of the Treatment Credibility Scale  MINI Mini-International Neuropsychiatric Interview  PSS-14 Perceived Stress Scale  SWLS Satisfaction with Life Scale  BDI-II Beck Depression Inventory-II  Montgomery Asberg Depression Rating Scale  BAI Beck Anxiety Inventory  SDS Sheehan Disability Scale  AAQ-7 Acceptance and Action Questionnaire  CFQ-7 Cognitive Fusion Questionnaire | Yes  NR  NR  Yes  Yes  Yes  Yes  Yes  Yes  Yes  Yes  Yes | NA | TSC adapted for autistic people,  Not reported for others. |  | **SC** |
| Pahnke et al., 2022 | **LR** | **SC** | **LR** | WAIS-R Wechsler Adult Intelligence Scale–Revised or  WAIS-III Wechsler Adult Intelligence Scale–Third Edition  CPT-II Conners’ Continuous Performance Test  D-KEFS Delis–Kaplan Executive Function System  MINI Mini-International Neuropsychiatric Interview  TCS Treatment Credibility Scale  PSS-14 Perceived stress scale PSS  SWLS Satisfaction with Life Scale  QOLI –Quality of Life Inventory  BDI- II Beck Depression Inventory–II  BAI Beck Anxiety Inventory  KSQ Karolinska Sleep Questionnaire  SDS Sheehan Disability Scale  AAQ-7 Acceptance and Action Questionnaire  CFQ- 7 Cognitive Fusion Questionnaire  CBAS Cognitive–Behavioral Avoidance Scale  SRS Social Responsiveness Scale  DEX-S Dysexecutive Questionnaire | Yes  Yes  NR  NR  Yes  Yes  Yes  Yes  Yes  Yes  Yes  Yes  Yes  Yes  Yes  Yes  Yes  Yes | NA | TSC adapted for autistic people  SRS – Yes  not reported for others. | HR  HR  HR  HR  HR  LR  HR  HR  HR  HR  HR  HR  HR  HR  HR  HR  LR  HR | **SC** |
| Quadt et al., 2021 | **SC** | **LR** | **SC** | MINI Mini International Neuropsychiatric Interview (Section O: Generalized Anxiety Disorders)  STAI-T Spielberger State and Trait Anxiety Inventory  STAI-S State anxiety score  BPQ Porges Body Perception Questionnaire  GAD-7 Generalized Anxiety Symptoms  MAIA Multidimensional Assessment of Interoceptive Awareness  TAS-20 Toronto Alexithymia Scale  PANAS Positive and Negative Affect Scale  PHQ-9 Patient Health Questionnaire | NR | NA | NR | HR  HR  HR  HR  HR  HR  HR  HR  HR | **HR** |
| Quist et al., 2015 | **LR** | **HR** | **SC** | PIRS-20 Pittsburgh Insomnia Rating Scale | NR | NA | NR | HR | **HR** |
| Ritschel et al., 2021 | **SC** | **SC** | **SC** | PPVT-4 Peabody Picture Vocabulary Test fourth edition  Wechsler Abbreviated Scale of Intelligence second edition  PSQ Participant Satisfaction Questionnaire with wellbeing questions developed for the intervention | NR | NA | Specifically developed Participant Satisfaction Questionnaire. | HR | **HR** |
| Russell et al., 2019 | **LR** | **LR** | **LR** | PHQ-9 Patient Health Questionnaire-9 items  CIS-R Clinical Interview Schedule-Revised (CIS-R)  BDI-II Beck Depression Inventory-II  SIGH-D (HAM-D) Structured Interview Guide for Hamilton Depression Rating Scale  GAD-7 Generalised Anxiety Disorder-7  OCI-R Obsessive–Compulsive Inventory-Revised  PANAS Positive and Negative Affect Schedule  WSAS Work and Social Adjustment Scale  SF-12 Short Form questionnaire-12 items  EQ-5D-5L EuroQol-5 Dimensions, five-level version  RBQ-2A Adult Repetitive Behaviour Questionnaire-2  RRQ Rumination–Reflection Questionnaire  Participant Global Rating of Change | Yes for all | NA | Unknown  NR  Yes  Unknown  Unknown  Yes  No  Used previously  Unknown  NR  Used previously  NR  NR | HR  HR  LR  HR  HR  LR  HR  SC  HR  HR  SC  HR  HR | **SC** |
| Sizoo & Kuiper, 2017 | **LR** | **SC** | **LR** | WAIS IV Wechsler Adult Intelligence Scale  HADS Hospital Anxiety and Depression Scale  GMS Dutch Global Mood scale  SRS-A Social Responsiveness Scale, Autism  RRQ Rumination-Reflection Questionnaire | NR  Yes  Yes  NR  NR | NA | NR  Used previously  Used previously  Yes  Used previously | HR  SC  SC  LR  SC | **SC** |
| Spain et al., 2017 | **LR** | **LR** | **LR** | TAS-20 Toronto alexithymia scale  Rosenberg self-esteem scale  Satisfaction with friendships questionnaire  LSAS Liebowitz Social Anxiety Scale  HADS Hospital Anxiety and Depression Scale  WSAS Work and Social Adjustment Scale  Satisfaction with friendships questionnaire developed specifically for  intervention study | NR  NR  No  NR  Yes  Yes  NA | NA | NR  NR  NR  Used previously  Used previously  Used occasionally  NA | HR  HR  HR  SC  SC  SC  HR | **SC** |
| Spek et al., 2013 | **LR** | **SC** | **LR** | WAIS-III Wechsler Adult Intelligence Scale  SCL-90-R Symptom Checklist-90-revised  RRQ Rumination-Reflection Questionnaire  GMS Dutch Global Mood Scale | Yes  Yes  Yes  Yes | NA | NR | HR  HR  HR  HR | **HR** |
| Tchanturia et al., 2016 | **SC** | **HR** | **LR** | DFlex Detail and flexibility questionnaire  MR Motivational Ruler *used successfully in previous evaluations  Patient feedback questionnaire | Yes  NR*  NA | **SC** | NR | HR  HR  HR | **HR** |
| Tsuchiyagaito et al., 2017 | **SC** | **SC** | **LR** | SCID-I Structured Clinical Interview for DSM-IV Axis I Disorders  WAIS-III Wechsler Adult Intelligence Scale  Y-BOCS Yale–Brown Obsessive–Compulsive Scale  PHQ-9 Patient Health Questionnaire-9  GAD-7 Generalized Anxiety Disorder-7  SDS Sheehan Disability Scale | NR | **HR** | NR | HR  HR  HR  HR  HR  HR | **HR** |
| Walhout et al., 2022 | **SC** | **SC** | **LR** | MATE 2.1 & Q1 Measurement of addiction for triage and evaluation  The Utrecht Coping List  MHRM The Mental Health Recovery Measure Dutch Version  RRQ Rumination Reflection Questionnaire Dutch version  DASS-21 Depression Anxiety Stress Scales | NR  Yes  Yes  Yes  Yes | NA | NR | HR  HR  HR  HR  HR | **HR** |
| Watanabe, 2021 | **SC** | **LR** | **LR** | QIDS-SR-J Quick Inventory of Depressive Symptomatology Self Report-  Japanese  CFQ-7- J Cognitive Fusion Questionnaire-7 Japanese  MAAS-J Mindful Attention Awareness Scale-Japanese  VQ-J Valuing Questionnaire-Japanese | Yes  Yes  Yes  Yes | NA | NR | HR  HR  HR  HR | **HR** |
| Backman et al., 2018 | **LR** | **SC** | **LR** | MADRS-S Montgomery and Asberg Depression Rating Scale  WHODAS 2.0, ‘Disability Assessment Schedule II  TCS Treatment Credibility Scale  ASD-Quiz (specific to intervention)  HADS Hospital Anxiety and Depression Scale  AAQ Acceptance and Action Questionnaire  SWLS Satisfaction with Life Scale | NR | NA | NR | HR  HR  HR  HR  HR  HR  HR | **HR** |
| Bemmer et al., 2021 | **SC** | **SC** | **LR** | WTAR Wechsler Test of Adult Reading  LSAS-SR Liebowitz Social Anxiety Scale—Self-Report  SRS-2 Social Responsiveness Scale-2—Adult Self-Report  DASS-21 Depression Anxiety Stress Scales  K10 Kessler Psychological Distress Scale  SIAS Social Interaction Anxiety Scale  SPS Social Phobia Scale | NR  Yes  Yes  NR  Yes  NR  NR | NA | NR  Commonly used  Yes  Yes  Used previously  Yes  Yes | HR  SC  LR  LR  SC  LR  LR | **SC** |
| Brandsma et al., 2022 | **LR** | **SC** | **LR** | KIDSCREEN-27 (Parent Report for QoL)  WHOQOL Quality of Life-Bref, psychological wellbeing subscale  SCL-90 Symptom Check-list-90  SPPA Self-perception Profile for Adolescents Dutch Version, self-esteem  subscale CBSA *Competentie Belevings schaal voor Adolescenten*  UGDS Utrecht Gender Dysphoria Scale, either Male-to-Female or  Female-to-Male scale.  SRS- A Social Responsiveness Scale for adolescents | NR  Yes  Yes  Yes  Yes  Yes | NA | Kidscreen (parent) used in other research with autistic children  NR for other measures | SC  HR  HR  HR  HR  HR | **HR** |
| Capriola-Hall et al., 2021 | **LR** | **SC** | **SC** | WASI-II Wechsler Abbreviated Scale of Intelligence-Second Edition  ASR Adult Self Report  UCLALS UCLA Loneliness Scale  DERS Difficulties in Emotion Regulation Scale  AIR‐SD American Institutes for Research Self Determination Scale | NR  Yes  Yes  Yes  Yes | NA | NR  NR  Used previously  Used previously  NR | HR  HR  SC  SC  HR | **SC** |
| Carey et al., 2022 | **SC** | **SC** | **LR** | ASC-ASD Anxiety Scale for Children for ASD  (completed by parents/teachers) | NA | NA | Yes  (for children) | LR | **SC** |
| Connor et al., 2020 | **SC** | **LR** | **LR** | WASI-2 Wechsler Abbreviated Scale of Intelligence: Second Edition  WJ-III ACH Woodcock Johnson Tests of Achievement: Third Edition  SRS-2 Social Responsiveness Scale, Second Edition  SFQ Social Functioning Questionnaire  GSE General Self-Efficacy Scale  PESE Perceived Empathic Self-Efficacy Scale  PSSE Perceived Social Self- Efficacy Scale  PHQ-9 Patient Health Questionnaire-9  GAD-7 General Anxiety Disorder Questionnaire-7, | Yes  Yes  Yes  Yes  Yes  Yes  Yes  Yes  Yes | NA | Not able to use validated given scarcity of measures for autistic people | HR  HR  HR  HR  HR  HR  HR  HR  HR | **HR** |
| de Bruin et al., 2015 | **SC** | **SC** | **LR** | AQ Autism Questionnaire  MAAS-A Mindful Attention and Awareness Scale–Adolescent version  PSWQ Penn State Worry Questionnaire  RRS Ruminative Response Scale  WHO-5 World Health Organization–Five Well-Being Index  SRS Social Responsiveness Scale | NR | NA | NR | HR  HR  HR  HR  HR  HR | **HR** |
| Ehrenreich-May et al., 2020 | **LR** | **SC** | **SC** | ADIS-IV-C/P Anxiety Disorders Interview Schedule for DSM-IV, Child Version  RCADS-P Revised Child Anxiety and Depression Scale–Parent Version  BASC-2-PRS-A Behavior Assessment System for Children, Second Edition  Parent Rating Scale–Adolescent Version  BASC-2-SRP-A Parent Rating Scale–Adolescent Version, and Self-Report  of Personality–Adolescent Version  SRS Social Responsiveness Scale  SCQ Social Communication Questionnaire | Yes  Yes  Yes  Yes  Yes  Yes | NA | NR | HR  HR  HR  HR  HR  HR | **HR** |
| Ekman & Hiltunen, 2015 | **LR** | **SC** | **LR** | GAF Global Function Rating scale | NR | NA | NR | HR | **HR** |
| Jackson et al., 2022 | **LR** | **SC** | **LR** | BASC-3 Behavioral Assessment System for Children, Scales used:  Anxiety, Attention Problems, Emotional Self-Control, Depression,  Hyperactivity, Behavioral Symptom Index, Externalizing Symptom  Index, and Internalizing Symptom Index  CSHQ Children’s Sleep Habits Questionnaire, Domain scales used: Sleep Duration, Sleep Onset Delay, and Daytime Sleepiness, and Total Score.  SRS-2 Social Responsiveness Scale  * Validated for younger age range than used in study | Yes*  Yes*  NR | NA | NR  NR  Yes* | HR  HR  HR | **HR** |
| Kemeny et al., 2022 | **SC** | **LR** | **LR** | Cohen’s Perceived Stress Scale  SSS Stress Survey Schedule for Persons with Autism and Other  Developmental Disabilities  SRS-2 Social Responsiveness Scale | Yes  Yes  Yes | NA | NR  Yes  Yes | HR  LR  LR | **SC** |
| Mahler et al., 2022 | **LR** | **SC** | **LR** | BRIEF-2 Behavior Rating Inventory of Executive Function, second edition  CQIA-2 Caregiver Questionnaire for Interoceptive Awareness,2nd edition | Yes  No | NA | Yes  No | LR  HR | **SC** |
| McGillivray & Evert, 2014 | **LR** | **SC** | **LR** | DASS Depression Anxiety Stress Scales  ATQ Automatic Thoughts Questionnaire  ASSQ Anxious Self-Statements Questionnaire | Yes  NR  NR | NA | NR | HR  HR  HR | **HR** |
| Murphy et al., 2017 | **LR** | **LR** | **LR** | ADIS-C/P Anxiety Disorders Interview Schedule for Children/Parents  CASI-anx Child and Adolescent Symptom Inventory-4 ASD Anxiety Scale  SRS Social Responsiveness Scale  *agreement between clinically-trained raters of ADIS, has been found to be excellent for youth with ASD | NR  NA  NA | NA | Yes *  Yes  Yes  (for children or youths) | LR  LR  LR | **SC** |
| Murray et al., 2015 | **LR** | **SC** | **LR** | CY-BOC Children's Yale-Brown Obsessive–Compulsive Scale | Yes | **LR** | Yes  (for children) | LR | **SC** |
| Pahnke et al., 2014 | **LR** | **SC** | **LR** | Stress Survey Schedule for Autism and Other Developmental Disorders  SDQ Strengths and Difficulties Questionnaires  BYI Beck Youth Inventories, subscales for anxiety, depression and anger. | Yes  Yes  Yes | NA | Yes  NR  Yes | LR  HR  LR | **SC** |
| Reaven et al., 2012 | **LR** | **SC** | **LR** | WASI Wechsler Abbreviated Scales of Intelligence  SCARED Screening for Childhood Anxiety and Related Emotional Disorders  ADIS-C/P Anxiety Disorders Interview Schedule for DSM-IV, .Child and Parent Vrsn  CGIS-S Clinical Global Impression Scale-Severity ratings | Yes  NR  Yes  NR | NA | Yes  NR  Used previously  NR | LR  HR  SC  HR | **SC** |
| Ridderinkhof et al., 2018 | **SC** | **SC** | **LR** | SRS Social Responsiveness Scale, Dutch version  ASEBA Achenbach System of Empirically Based Assessment, Dutch version,  subscale attention problems, and the internalizing and externalizing problems broadband syndrome scales  CBCL Child Behavior Checklist  YSR Youth Self Report  RRS Ruminative Response Scale  CSQ-CA Chronic Stress Questionnaire for Children and Adolescents  CSRQ Chronic Sleep Reduction Questionnaire  WHO-5 World Health Organization- Five Well-Being Index  CAMM Children’s Acceptance and Mindfulness Measure, Dutch version | Various methods | NA | Yes  NR for  remaining | LR  HR  HR  HR  HR  HR  HR  HR  HR | **SC** |
| Ridderinkhof et al., 2021 | **SC** | **SC** | **LR** | ADIS-C/P Anxiety Disorders Interview Schedule for children  SCID Structured Clinical Interview for DSM-5 Disorders for Children  CBCL Child Behavior Check- list, broadband scale internalizing problems  YSR Youth Self Report  Wechsler Scale of Intelligence  GAS Goal Attainment Score (Individualised) | NR | NA | NR | HR  HR  HR  HR  HR  HR | **HR** |
| Russell et al., 2013 | **SC** | **SC** | **LR** | YBOCS Yale–Brown Obsessive Compulsive Scale  MINI 5.0 Neuropsychiatric interview  YBOCS-Symptom Checklist  D-YBOCS Dimensional YBOCS  CGI Clinical Global Impression  CGIi CGI improvement  OCI-R Obsessive Compulsive Inventory-Revised  BDI Beck Depression Inventory  BAI Beck Anxiety Inventory  LSAS Liebowitz Social Anxiety Scale  WSAS Work and Social Adjustment Scale Youth  SCAS Spence Children’s Anxiety Scale  PR-CHOCI-R Children’s Obsessive Compulsive Inventory-Parent Version  FAS-PR Family Accommodation Scale-Parent Report | Yes  NR for  others | NA | NR | HR  HR  HR  HR  HR  HR  HR  HR  HR  HR  HR  HR  HR  HR | **HR** |
| Salem-Guirgis et al., 2019 | **LR** | **SC** | **LR** | SCQ- Social Communication Questionnaire – Lifetime Version  SRS-2 Social Responsiveness Scale, Second Edition  WASI-II Wechsler Abbreviated Scale of Intelligence-II  BASC-2 Behavior Assessment System for Children, Second Edition  BASC-2 PRS Parent Rating Scale  BASC-2 SRP Self-Report of Personality  RRS Ruminative Response Scale  ERC Emotion Regulation Checklist  ERQ-CA Emotion Regulation Questionnaire-Child  CAMM Youth Mindfulness The Child and Adolescent Mindfulness Measure | Various | NA | Yes  Used previously  NR  Used previously  Used previously  NR  NR  NR  NR  Used previously | LR  SC  HR  SC  SC  HR  HR  HR  HR  SC | **SC** |
| Strang et al., 2021 | **SC** | **SC**  . | **LR** | Exploratory qualitative study: Framework analysis identified 11 Resulting Clinical Approaches | NR | NA | Specifically developed review Questionnaire. | HR | **HR** |
| van Steensel et al., 2014 | **LR** | **SC** | **LR** | ADIS-C/P Anxiety Disorder Interview Schedule-Child/Parent version  EQ-5D EuroQol-5D | Yes  Yes | NA | Used previously  (with children)  Used previously  (with children) | SC  SC | **SC** |
| Wise et al., 2019 | **SC** | **LR** | **LR** | WASI-II Wechsler Abbreviated Scale of Intelligence- Second Edition  ADIS-5 The Anxiety Disorders Interview Schedule for DSM-5  HAM-A The Hamilton Anxiety Scale  HAM-D The Hamilton Depression Scale  CGI-S The Clinical Global Impression-Severity  CGI-I The Clinical Global Impression-Improvement  BAI The Beck Anxiety Inventory | NR  NR  Yes  NR  NR  NR  NR | NA | NR | HR  HR  HR  HR  HR  HR  HR | **HR** |
| Wolters et al., 2016 | **LR** | **SC** | **LR** | CY-BOCS Children's Yale-Brown Obsessive Compulsive Scale  ADIS-C/P Anxiety Disorder Interview Schedule for DSM-IV Child/Parent Version  WISC-III Wechsler Intelligence Scale for Children  CSBQ Children's Social Behavior Questionnaire | Yes  Yes  Yes  Yes | **LR** | NR  NR  NR  Yes - validated | HR  HR  HR  LR | **SC** |
| Yang & Chung, 2022 | **LR** | **SC** | **LR** | KWAIS‐IV Korean‐WAIS  AQ Autism Spectrum Quotient (Korean Version)  STAI State‐Trait Anxiety Inventory (Korean Version)  ATQ‐N Automatic Thought Questionnaire‐Negative (Korean Version)  PANAS Positive and Negative Affect Schedule (Korean Version)  ABC Aberrant Behavior Checklist (Korean Version)  MARS A modified version of the mobile app rating scale  Antecedent Behavior Consequence Direct Observation | Yes  Yes  Yes  Yes  Yes  Yes  Yes | NA | Yes  NR for  others | LR  HR  HR  HR  HR  HR  HR | **SC** |
| Zaharia et al., 2021 | **SC** | **SC** | **SC** | Attentional deployment/ Suppression Questionnaire, Attentional  Deployment subscale  Emotion Regulation Questionnaire, Cognitive Reappraisal subscale  Response Modulation subscale from the Berkeley Expressivity  Questionnaire, Emotional Expressiveness Questionnaire and two new  specific items assessing positive response modulation. | NR | NA | NR | HR  HR  HR | **HR** |

RoB: Risk of bias; AO: Adult only participants; AC Adults plus child participants; HR: High risk; SC: Some concerns; LR: Low risk; NA: risk of bias not applicable for study; NR: not reported.
