# Supplementary material for: Fit-for-purpose Psychological Interventions to Support the Well-Being of Autistic Adults: A Systematic Review
Source: Autism Dev Lang Impair. 2026 May 15;11:23969415261436238. doi: 10.1177/23969415261436238 (PMC13180214; doi:10.1177/23969415261436238)
Supplement: sj-docx-5-dli-10.1177_23969415261436238 - Supplemental material for Fit-for-purpose Psychological Interventions to Support the Well-Being of Autistic Adults: A Systematic Review [file sj-docx-5-dli-10.1177_23969415261436238.docx]

Supplemental Information 5

Candidate mechanisms of change and the nature of the evidence provided within studies

| **Candidate mechanism of change arguments** | **Nature of evidence** |
| --- | --- |
| **Improved emotion regulation skills**  Approaches to changing Emotion Regulation (ER) skills and included: 1) mindfulness and acceptance-based techniques, 2) increasing interoceptive awareness 3) psychoeducation and 4) increasing psychological flexibility. Various outcome measures were used for ER skills, e.g. the *Difficulties in Emotion Regulation scale* or the *Coping Inventory for Stressful Situations.* | Statistical and qualitative reporting, predominantly from non-RCT studies. Beck et al., 2020; Bemmouna et al., 2022; Brezis et al., 2021; Hare et al., 2016; Kuroda et al., 2022; Lawson et al., 2022; Pahnke et al., 2019; Pahnke et al., 2022; Quadt et al., 2021; Sizoo & Kuiper, 2017; Tchanturia et al., 2016. |
| **Reduced rumination tendencies to reduce symptoms of anxiety and depression**  Mediation analyses suggested a decline in rumination tendencies was positively related to the effect of the mindfulness-based protocol on the reduction in symptoms of anxiety and depression. Rumination is described as the tendency to think repetitively about the causes, situational factors and consequences of one’s emotional experience (Nolen-Hoeksema et al., 2008). | One study had no comparison group.  (RCT) Spek et al., 2013 *(n*=41);  (QE) Kiep et al., 2015 (*n*=50)  (data for 20 participants was used in both studies) |
| **Increased cognitive defusion skills to reduce the impact of emotional distress in the moment.**  A brief (5-minute) cognitive defusion or cognitive distraction intervention were similarly effective in immediately reducing thought believability and thought discomfort in the autistic and non-autistic participant groups, with moderate to large effect sizes | Statistical testing at a single timepoint.  (RCT) Maisel et al., 2019 (*n*=68) |
| **Reduction in insomnia symptoms toward reduced symptoms of anxiety and depression.**  Insomnia is a transdiagnostic process associated with the onset and maintenance of mental health difficulties (Dolsen et al., 2014 as cited in Lawson et al., 2022). The outcome measures used were: *Insomnia Severity Index* and/or the *Pittsburgh Sleep Quality Index*. | Reported significant improvement in sleep quality outcome measures in three studies with no comparison groups.  (QE) Lawson et al., 2022;  (QE) Ishii et al., 2022;  (QE) Quist et al., 2015 |
| **Contribution of positive social support experienced in group therapy to perceived Quality of Life (QoL) outcomes.**  Two studies had active comparison arms that provided group social interaction thought to be equivalent to the active conditions. Authors concluded that the benefits may be due to the group settings themselves as opposed to the specific protocol techniques. | Statistical testing of intervention outcomes (QoL), not of the contribution of social support as a mechanism.  (RCT) Braden et al., 2022;  (RCT) Hesselmark et al., 2014 |
| **Positive therapeutic alliance.**  The importance of the therapeutic alliance was discussed within three studies but not as a candidate mechanism of change. | Qualitative reporting in non-RCT based studies.  (Case) Dandil et al., 2020;  (QE) Helverschou et al., 2019;  (QE) Lobregt-van Buuren et al., 2019 |

RCT: randomised control trial; QE: quasi-experimental.
